# Natural interviewing equilibria in matching settings

**DOI:** 10.1007/s00355-024-01541-2

**Published:** 2024-08-30

**Authors:** Allan Borodin, Joanna Drummond, Kate Larson, Omer Lev

**Affiliations:** 1 Department of Computer Science, University of Toronto, 10 King’s College Road, Toronto, ON M5S 3G4 Canada; 2 Cheriton School of Computer Science, University of Waterloo, Waterloo, N2L 3G1 ON Canada; 3 Department of Industrial Engineering and Management, Ben-Gurion University of the Negev, 1 Ben-Gurion Blvd., Beersheba, 8410501 Israel

## Abstract

A common assumption in matching markets is that both sides fully know their preferences. However, when there are many participants this may be neither realistic nor feasible. Instead, agents may have some partial (perhaps stochastic) information about alternatives and will invest time and resources to better understand the inherent benefits and tradeoffs of different choices. Using the framework of matching medical residents with hospital programs, we study strategic behaviour by residents in a setting where hospitals maintain a publicly known master list of residents (i.e., all hospitals have an identical ranking of all the residents, for example, based on grades) and residents have to decide with which hospitals to interview, before submitting their preferences to the matching mechanism. We first show the existence of pure strategy equilibrium under very general conditions. We then study the setting when residents’ preferences are drawn from a known Mallows distribution. We prove that assortative equilibrium (*k* top residents interview with *k* top hospitals, etc.) arises only when residents interview with a small number of programs. Surprisingly, such equilibria (or even weaker notions of assortative interviewing) do not exist when residents can interview with many hospital programs, even when residents’ preferences are very similar. Simulations on possible outcome equilibrium indicate that some residents will pursue a reach/safety strategy.

## Introduction

Since Gale and Shapley’s groundbreaking work (Gale and Shapley 1962), the use of stable matching mechanisms has proliferated across numerous domains. Applications range from matching children to schools to matching refugees to countries (Andersson 2019). A central goal of these mechanisms is to ensure that participants (or agents) have no incentive to try to manipulate the final outcome of the matching process by being strategic about the choices they make and actions they take. However, when deployed in practice, many of the assumptions behind the Gale-Shapley (deferred-acceptance) algorithm no longer hold. For example, agents may have partial preferences or ties (Drummond and Boutilier 2014; Rastegari et al 2013; Irving et al 2009), quotas imposed on matching outcomes (Goto et al 2016), distributional constraints (Kurata et al 2017), and computational constraints, for which compact representations of preferences are useful (e.g., Gelain et al 2009; Pini et al 2014).

One real-world domain where matching mechanisms are implemented is medical residencies (Roth 2002). In many countries, such as Canada and the United States, medical students are assigned to hospital residencies through matching mechanisms. For example, the National Residency Matching Program (NRMP), an American program for matching medical residents to hospitals, in 2023 offered 37,425 positions for first-year residents for 5487 hospital programs (National Resident Matching Program 2023). In this paper, we focus on a problem that arises in medical residency matching settings, where the number of options for residents is large. As noted above, in the NRMP residents need to choose from over 5000 positions, yet they apply to only eleven on average (Anderson et al 2000). Furthermore, residents are often faced with significant uncertainty regarding which hospital may be the best match for themselves. While there are publicly available rankings for hospitals, an individual’s preferences will also be influenced by specific, personal considerations (e.g., the personal chemistry with the people in the hospital). One way to address this uncertainty is to *interview* with a set of hospitals; this allows the resident to understand and refine their personal ranking between the possible hospitals. However, this requires the resident to choose the set of hospitals they will interview, based on the limited information they have.

The problem of selecting an appropriate interviewing set gives rise to new strategic concerns and widely used mechanisms—which are strategyproof when assuming every resident initially knows their full ranking of the hospitals—are no longer strategyproof (Haeringer and Klijn 2009; Calsamiglia et al 2010). This observation, that interview-set selection is a strategic decision, motivates our work. Inspired by the medical residencies matching problem, we analyze the Nash equilibrium strategies that arise when residents must select interview sets, knowing that a widely used matching mechanism, Resident-Proposing Deferred Acceptance (rp-da), will be used. In particular, we examine the possibility of *assortative* equilibria, in which residents are divided into groups, with each group interviewing in the same sets of hospitals.

We provide an example to help illustrate some of the strategic reasoning which arises when residents must select interview sets.

### Example 1

Suppose we have 4 hospitals—$h_{1},h_{2},h_{3},h_{4}$, and 4 residents—$r_{1},r_{2},r_{3},r_{4}$. All hospitals know the residents’ quality ($r_{1}$ being the best, followed by $r_{2}$, then $r_{3}$, and $r_{4}$ is the worst), and every resident knows their position in the hospitals’ ranking. Suppose residents have two possible rankings of hospitals: with probability 0.5 a resident’s ranking of hospitals is $h_{1}\succ h_{2}\succ h_{3}\succ h_{4}$, and with probability 0.5, it is $h_{2}\succ h_{1}\succ h_{4}\succ h_{3}$. Assume that residents can only interview with at most 2 hospitals.

Residents $r_{1}$ and $r_{2}$ can choose to interview at $h_{1}$ and $h_{2}$, while residents $r_{3}$ and $r_{4}$ can choose to interview at hospitals $h_{3}$ and $h_{4}$. Such a choice is both assortative and stable—both $r_{1}$ and $r_{2}$ know they will never prefer $h_{3}$ and $h_{4}$ over the hospitals they interview with; and because of this, both $r_{3}$ and $r_{4}$ know hospitals $h_{1}$ and $h_{2}$ will surely be taken already by the time it is their turn to interview, so no point in interviewing there. In this case, there is no other equilibrium.

If the probability of any ordering is as likely as any other, then many other interviewing strategies are stable, including non-assortative ones. For example, $r_{1}$ and $r_{3}$ interviewing at $h_{2}$ and $h_{4}$ while $r_{2}$ and $r_{4}$ interview at $h_{1}$ and $h_{3}$.

### Our contributions

Under the assumption that hospitals maintain a common master list over residents (e.g. GPAs, exam results, etc. (Hafalir 2008; Zhu 2014; Chen and Pereyra 2015; Ajayi 2011)), we explore the structure of Nash equilibrium when residents are required to select *k* hospitals with which to interview (and, thus, rank for the matching mechanism). We show the following:

- A pure strategy Nash equilibrium exists for this game.
- Using the Mallow’s model for sampling resident preferences, an *assortative* equilibrium exists for very small interviewing sets. This equilibrium is “natural” in that hospitals and residents are stratified: highly ranked residents interview at well-regarded hospitals; medium residents interview at medium hospitals; and low-ranked residents interview at low-ranked hospitals. Indeed, we initially believed this would be the natural equilibria in most cases (as Ajayi 2011 seemed to indicate).
- However, in the Mallow’s model, if residents use larger interview sets, assortative strategies no longer form an equilibrium. That said, new equilibria appear, where residents select interview sets that contain both “reach” and “safety” alternatives.

We note that throughout the paper we use the terminology of hospitals and residents, but emphasize that this is merely for clarity and consistency. Our results hold for any setting in which one side cannot provide a full ranking of the other, and must decide how to focus its attention so as to learn more about particular alternatives. Such scenarios could include students interviewing at schools, universities, and recruiting faculty candidates, among others.

## Related research

While there is a rich literature on matching markets and stability (e.g. Gusfield and Irving 1989), the importance and impact of interviews in these markets is not as well understood. In recent work (Echenique et al 2022), through analysis of the NRMP, observed that most doctors match with one of their most preferred internship programs, despite having very similar preferences. They argued that this apparent contradiction is an artifact of the interview process that precedes the match. These findings highlight the importance of better understanding market interactions, including interviews, that happen before (and after) the actual market. A similar lesson can be taken from Harless and Manjunath (2018) work that studied the impact of the allocation rule (e.g. matching mechanism) on the interviewing process, illustrating how these two steps influence each other. Indeed, several works on interviewing—using various models with varying similarity to ours—have examined the equilibria that arises when assuming the existence of interviews.

One thread of research has studied interviewing policies that aim to minimize the total number of interviews conducted while also ensuring stability in the final match. For example, Rastegari et al (2013) showed that while finding the minimal interviewing policy is NP-hard in general, there are special cases where a polynomial-time algorithm exists, while Drummond and Boutilier (2014) approached the problem using the framework of minimax regret and proposed heuristic approaches for interviewing policies. These papers assume that interview policies are implemented centrally, ignoring the situation where agents may choose with whom to interview, whereas our work explicitly studies strategic issues arising from situations where agents choose their interview strategies.

There is a body of literature that addresses strategic interviewing in matching markets, but many of the papers ask different questions or make different modelling assumptions than we do in our work. Manjunath and Morill (2023) studied the problem of “interview hoarding” where one side of the market (e.g. residents) can interview as many candidates as they wish, while the other side (e.g. hospitals) are limited in the number of interviews they may conduct. The authors show that this leads to problematic outcomes compared to settings where interviews are limited on both sides. In particular, no resident that would have been matched in the setting with limited interviews is better off in the unlimited setting and many residents are worse off. This is consistent with Kadam (2015) findings that relaxing residents’ interview constraints can adversely impact lower-ranked residents. These results support our modelling choice of restricting the size of agents’ interview sets. We note, however, that research has also shown that limiting the size of agents’ interview sets may have strategic implications. For example, in two related papers, Haeringer and Klijn (2009) and Calsamiglia et al (2010) show that limiting the number of interviews an agent may partake in can lead to less stability in the market and encourage agents to misreport their preferences, while He and Magnac (2017) showed, empirically, that imposing an interviewing cost may lead to decreased match quality. Because of these findings, we make no claim that we are using “optimal” interview set sizes, and we consider the question of the size of interview sets to be outside the scope of this paper.

Several other authors have also studied matching markets with limited/fixed interview sets (Immorlica and Mahdian 2005; Beyhaghi et al 2017; Beyhaghi and Tardos 2019). Unlike our work, these papers typically assume uncorrelated preferences (i.e. every hospital independently ranks residents idiosyncratically), allow for a fixed probability of selection (Immorlica and Mahdian 2005), or uniform preferences within subgroups of residents/hospitals (Beyhaghi et al 2017). We believe that these modelling assumptions are overly strong and empirical evidence indicates that preferences are correlated (e.g. Echenique et al 2022), which we try to capture in our preference models.

We are particularly interested in what we call “natural” interviewing equilibria. These equilibria are assortative in that top residents interview with (and are matched with) top hospitals while bottom-ranked residents interview with (and are matched with) bottom-ranked hospitals etc. Lee (2017) showed the existence of such equilibria in matching markets. Their results, however, relied on several strong assumptions including a large market assumption and strong restrictions on the preference models. We are interested in understanding whether it is possible to support assortative equilibria under broader assumptions. Other papers (e.g. Chade and Smith 2006; Chade et al 2014; Ali and Shorrer 2023), motivated by the college-selection problem, have also studied the structure of the resulting equilibria. While Chade and Smith (2006) showed that students would greedily select which colleges to interview with under the assumption that admissions prospects across colleges were stochastically independent, Ali and Shorrer (2023) argued that changes in the underlying model, namely allowing for correlations, results in equilibrium outcomes where students apply for both “reach” and “safety” colleges. While the problem we study is very different since we assume a centralized matching market while these papers study a decentralized process, we find these papers relevant and informative as they hint at the importance of correlated preferences for students/residents in whether assortative or “reach and safety” strategies form equilibria.

Finally, we mention the work of Lee and Schwarz (2017). They studied a multi-stage worker-firm game where one side of the market (e.g. firms) had to first, at some fixed cost, select workers with which to interview before proceeding to a centralized matching market running (firm-proposing) deferred acceptance. Their key finding was if there was no coordination, then all firms were best off each picking *k* random workers to interview. However, if firms could coordinate then it was best for them to each select *k* workers so that there was perfect overlap (forming a set of disconnected complete bipartite interviewing subgraphs), i.e., an assortative equilibirum. This finding, while very elegant, relies heavily on the assumption that all firms and workers are *ex-ante* homogeneous, with agents’ revealed preferences being idiosyncratic and independent. In particular, for the results to hold either agents have effectively no information about their preferences before they interview, or the market must be perfectly decomposable into homogeneous sub-markets that are known before the interviewing process starts In this paper, we study a similar, multi-stage game, but we relax these assumptions. Instead, we assume that agents’ preferences are correlated and make no assumptions about the decomposability of the market into sub-markets.^1^ We are interested in understanding and characterizing the resulting equilibrium outcomes, providing insights into how sensitive so called “natural” equilibria are to the underlying preference structures of the agents.

## An interviewing game with limited interviews

Using the resident-hospital matching problem as our basic framework, we assume there is a set, $R=\{r_1,\ldots , r_n\}$ of residents and a set, $H=\{h_1,\ldots , h_n\}$ of hospitals.^2^ Every $h_i\in H$ has (strict) preferences over *R*, and every $r_i\in R$ has (strict) preferences over *H*. These are represented by $H_\succ = \{\succ _{h_1}, \ldots , \succ _{h_n}\}$ and $R_\succ =\{\succ _{r_1},\ldots , \succ _{r_n}\}$ respectively.

We are interested in *one-to-one matchings*; residents can only do their residency at a single hospital, and hospital programs can accept at most one resident. A *matching* is a 1–1 function $\mu : R \cup H \rightarrow R \cup H$, such that $\forall r \in R$, $\mu (r) \in H\cup \{r\}$, and $\forall h\in H$, $\mu (h) \in R \cup \{h\}$. If $\mu (r)=r$ or $\mu (h)=h$ then we say that *r* or *h* is unmatched. We assume that residents prefer to be assigned to any hospital over not being matched, and hospitals prefer to have any resident over not filling the position. A matching $\mu$ is *stable* if there does not exist some $(r,h) \in R\times H$, such that $h \succ _r \mu (r)$ and $r \succ _h \mu (h)$.

Critically, we assume the existence of a *master list*, $\succ _{ML}$, over residents, which is shared by all $h\in H$, such that $\succ _{h}=\succ _{ML}$. This assumption implies that all hospitals share identical preferences over residents. This captures scenarios such as when grades or GPAs are used to rank residents, or when residents are required to write standardized exams (Irving et al 2008; Chen and Pereyra 2015; Zhu 2014)). Without loss of generality, $r_i\succ _{ML}r_{i+1}$, $\forall i< n$. We further assume that every $r\in R$ is aware of their ranking on this master list.

Residents, on the other hand, have idiosyncratic preferences over hospitals. This may be based on, for example, location, potential colleagues, career opportunities for partners, *etc*. In particular, we assume there is some underlying, commonly known, preference distribution, $D$, from which each $r\in R$ draws $\succ _r$ independently. If resident *r* draws preference ranking $\eta$ from $D$, then $h_i \succ _{\eta } h_j$ means that $h_i$ is preferred to $h_j$ by *r* under $\eta$.

Critical to our model is the assumption that residents do not initially know their true preferences, but *refine* their information by conducting *interviews* with hospitals. If a resident was able to interview every hospital in *H* then they would know their true preferences. However, this is infeasible and instead, each resident has an *interviewing budget* of $k<n$ hospitals. Let $I(r)\subseteq H$, $|I(r)|\le k$ be resident *r*’s interview set, consisting of the set of hospitals *r* has selected to interview. Once the interviews are completed, *r* knows its preferences over $h\in I(r)$, though does not necessarily have any additional information over $h'\in H\setminus I(r)$.

Once all residents have interviewed with their selected hospitals, they enter into a matching process, using the preference information obtained through their interviews. In this paper, we use the standard resident-proposing deferred acceptance (rp-da). The resulting matching, $\mu$, is guaranteed to be stable, resident-optimal, and hospital-pessimal (Gale and Shapley 1962). This stable matching is also guaranteed to be unique, as stable matching problems with master lists have unique stable solutions (Irving et al 2008). Thus our results directly hold for any mechanism that returns a stable matching, including hospital-proposing deferred acceptance and the greedy linear-time algorithm (Irving et al 2008).

We summarize our model and assumptions for the interviewing game in which residents engage. We call this game the **Interviewing Game with Limited Quotas**, or **ILQ**.

- Each $r\in R$ and $h\in H$ is informed of the master list $\succ _{ML}$.
- Each resident $r \in R$ simultaneously selects an interviewing set $I(r)\subset H$, $|I(r)|\le k$.
- Each resident $r\in R$ interviews with hospitals in *I*(*r*) and learn their own preference, $\succ _{r|I(r)}$ over members of *I*(*r*).
- A central matching system runs resident-proposing deferred acceptance (rp-da) using $\succ _{ML}$ as the preference for all $h\in H$, and $\succ _{r|I(r)}$ for all $r\in R$. Any hospital $h\not \in I(r)$ is reported to be unacceptable by *r*.

### Utility functions for the interviewing game

We require a clear specification of the residents’ utility functions, as this supports the choices they make when deciding which hospitals to interview. Thus, in this subsection, we describe how we derive principled utility functions for the residents, based on their preferences and the expected match.

We first assume that residents share some common scoring function, $v:H\times H_\succ \mapsto \mathbb {R}$ such that for any ranking over hospitals, $\eta \in H_\succ$, $h_i\succ _\eta h_j$ if and only if $v(h_i,\eta )>v(h_j, \eta )$. The existence of such a scoring function is used in other literature (e.g. Coles and Shorrer 2014) and allows for flexibility in the modelling of the problem. For now, we merely assume the existence of such a scoring function and will explore different instantiations later.

Second, we make the critical observation that a resident, $r\in R$, need only be concerned about other residents that are higher ranked in the master list, $\succ _{ML}$. If a lower ranked resident, $r'$, is matching to some $h\in I(r)$, then it must be the case the *r* is matched to some $h'$ such that $h'\succ _{r} h$ since otherwise the matching would be unstable.

This greatly simplifies the formulation of the utility function for a resident as we need only consider the interview sets of higher-ranked residents, its own choice with whom to interview, and the probability with which it has a particular preference ranking over hospitals.

We introduce notation to help support the development of the utility function for a resident. Consider some resident, $r_j$, and interview sets, $I(r_1),\ldots , I(r_{j-1})$, for all $r_i\succ _{ML} r_j$. Furthermore, define $m=\mu _{|r_1,\ldots , r_{j-1}}$ to be the partial matching that arises when rp-dc is run. The set of preferences that are consistent with this partial match is

$$\begin{aligned} T(r_j,m) =&\{\xi \in H_\succ | \exists I(r_{j}) {\text { s.t. }}\, \forall h' \in I(r_{j}) {\text { s.t. }}\, h' \succ _{\xi } m(r_j), \\&\exists r_a {\text { s.t. }}\, r_a \succ _{ML} r_j \wedge m(r_a) = h'\}. \end{aligned}$$

Observe that $T(r,m)\not = \emptyset$ for all $r\in R$, and that $T(r_1,m)=H_\succ$.

Given some preference distribution *D*, the probability that some particular partial match, $m'$, arises, given interview sets $I(r_1),\ldots , I(r_{j-1})$ is simply the probability that the residents had preferences consistent with $T(r_j,m'):$

$$\begin{aligned} P(m'|(I(r_i))_{i=1}^{j-1} ) = \prod _{i=1}^{j-1} \sum _{\xi \in T(r_i,m)} P(\xi | D). \end{aligned}$$

Now resident $r_j$ must determine the probability with which it will be matched to a particular hospital, *h*, since its utility is determined by how it perceives the program quality. We define

$$\begin{aligned} M^*(I(r_j), (I(r_i))_{i=1}^{j-1}, \eta , h) =&\{m | m(r_j) = h; \forall r_i\in \{r_1,\ldots ,r_{j-1} \}, m(r_i) \in I(r_i);\\&\forall x\! \in \!I(r_j), {\text { if }}\, x \succ _{\eta } h, \exists r_i {\text { s.t. }}\, x \!\in \!I(r_i) {\text { and }}\, m(r_i) \!=\! x\} \end{aligned}$$

to be the set of (partial) matches where $r_j$ is matched to hospital *h*, given interview sets for residents $r_1,\ldots , r_{j-1}$, interview set $I(r_j)$ for resident $r_j$ with preference ranking $\eta$. Since the preference rankings of residents $r_l$ such that $r_j\succ _{ML} r_l$ do not change what hospital $r_j$ is matched to, for any complete matching, $\mu$, we have

$$\begin{aligned} P(\mu (r_j) = h | \eta , I(r_j), (I(r_i))_{i=1}^{j-1}) = \sum _{m\in M^*(I(r_j), (I(r_i))_{i=1}^{j-1}, \eta , h)} P(m'|(I(r_i))_{i=1}^{j-1} ). \end{aligned}$$

Bringing everything together, the utility function for resident $r_j$, given its interview set, $I(r_j)$ is

1 $$\begin{aligned} u_{r_j}(I(r_j)) = \sum _{h\in I(r_j)} \sum _{\eta \in H_\succ } v(h,\eta ) P(\eta |D) P(\mu (r_j) = h | \eta , I(r_j), (I(r_i))_{i=1}^{j-1}). \end{aligned}$$

This utility function has an intuitive interpretation: it weights the value of a hospital by how likely the resident will be matched to it, given the interview-set choices of “more desirable” residents.

## Equilibria analysis: general results

We start our analysis by studying the most general form of the **ILQ**game possible. Recall that an **ILQ**game is defined as $\Psi = \langle n, k, D, v\rangle$ where $n=|R|=|H|$, *k* is the number of interviews any resident can conduct (also known as the quota), *D* is the underlying distribution from which residents’ preferences are being drawn, and *v* is the scoring function over hospitals that residents use. We start by placing no restrictions on the structure of the underlying preference rankings of the residents, nor do we place any constraints on their utility functions. Furthermore, to simplify notation we drop *n* from the **ILQ**notation unless it influences the results. We start by presenting an existence result, namely the existence of a pure strategy equilibrium for this game.^3^ We follow this by outlining general conditions under which this equilibrium might take a particularly appealing form, namely assortative interviewing. We then instantiate the residents’ preference models using a common probabilistic model for preferences (the $\phi$-Mallows model) and explore how this class of preference rankings support assortative interviewing.

### Theorem 1

Given any **ILQ**game $\Psi = \langle k, D, v\rangle$ with $k>0$, there exists a pure strategy equilibrium.

### Proof

We wish to show that if every resident chooses their expected utility-maximizing interviewing set, this results in an equilibrium. Given any resident $r_j$ who is *j*th in the hospitals’ rank- ordered list, $r_j$’s expected payoff function only depends on residents $r_1,\ldots ,r_{j-1}$. As $r_j$ knows that each other resident $r_i$ is drawing from distribution $D$ *i.i.d.*, they can calculate $r_1,\ldots ,r_{j-1}$’s expected utility maximizing interview set, using Eq. 1. Their payoff function depends only on $D$ and $I(r_1),\ldots ,I(r_{j-1})$, all of which they now have. They then calculate the expected payoff for each $n \atopwithdelims ()k$ potential interviewing sets, and interview with the one that maximizes their expected utility. Of course, when there are ties between the expected payoff of different strategies, multiple equilibria may arise. $\square$

Theorem 1 is an existence theorem. It does not provide any additional insight into the equilibrium behavior, nor does it provide any insight as to how this equilibria may be computed beyond a brute-force approach. This leads us to our next set of questions, namely under what conditions will a particular class of natural interviewing strategies form an equilibrium. We are particularly interested in *assortative interviewing strategies*.

### Definition 1

Given **ILQ**game $\Psi = \langle k, D, v\rangle$ with $k>0$, an interviewing strategy profile is *assortative* if and only if for $j = 0,1,2, \ldots , \frac{n}{k}-1$, each resident $r \in \{r_{jk+1},\ldots ,r_{jk+k}\}$ chooses to interview with the set of *k* hospitals $\{h_{jk+1},\ldots ,h_{jk+k}\}$.^4^

We view assortative strategies as being “natural” in that hospitals and residents are stratified: highly-ranked residents on the master list interview at well-regarded hospitals; mid-ranked residents interview at what they expect to be mid-ranked hospitals; and low-ranked residents interview at low-ranked hospitals. We start by deriving conditions that ensure assortative interviewing. We show that there exist scenarios in which one need only focus on the behavior of a single agent, namely $r_{k}$ where *k* is the interviewing budget. If assortative interviewing is a best response for resident $r_k$ when all other residents $i<k$ interview assortatively, then assortative interviewing is a best response for *every* resident $r_i$ ($i<k$) when all other residents interview assortatively. In other words, determining if assortative interviewing is a best response for $r_k$ is sufficient to show that assortative interviewing is a best response for the first *k* residents (and is thus an equilibrium for them in this game).

### Theorem 2

Let $\Psi =\langle k, D , v\rangle$ be an **ILQ**game with quota *k*, preference distribution $D$, and resident scoring function, *v*. Assume residents $r_1,\ldots , r_{k-1}$ all interview assortatively. Then, if resident $r_k$’s best response is to interview assortatively under this setting, it is a best response for any resident $r_1,\ldots ,r_{k}$ to interview assortatively. Moreover, this forms a unique best-response for $r_{1},\ldots ,r_{k}$.

### Proof

We introduce an indicator function to simplify notation for when a hospital is a resident’s top available choice. For any hospital *h* and agent *i*, let $b^{j}(h,\eta ) = 1$ if and only if *h* is available when $r_{j}$ makes their choice (i.e., $r_{1},\ldots ,r_{j-1}$ have not been allocated *h*), and is their most-desirable available alternative (i.e., $h \succ _\eta h'$ for all other $h'$ available); and 0 otherwise. Directly following from Eq. 1 the utility of resident $r_j$ when interviewing with hospitals $S\subset H$ is:

$$\begin{aligned} u_{r_j}(S)&= \sum _{h \in S} \sum _{\eta \in H_\succ } v(h,\eta )P(\eta , D )b^{j}(h,\eta ) \end{aligned}$$

Since for $r_{1}$, it is always true that $b^{1}(h,\eta ) = 1$ for any desired *h* (since $r_{1}$ goes first, no $h\in H$ has been allocated by another $r\in R$), suppose it will interview in a set of *k* hospitals $\{h_{1},\ldots ,h_{k}\}$ (the numbering according to $r_{1}$’s choices as determined by the distribution *D*). We are concerned with the best response strategy of $r_k$ which only depends on the strategies of $r_i$ for $i < k$. Suppose there is no assortative equilibrium, and let $r_{i}, i < k$, be the resident with the lowest index for which it is better off interviewing in set $S'\ne \{h_{1},\ldots ,h_{k}\}$. Then $b^{i}(h,\eta )\ge b^{k}(h,\eta )$, with the inequality being strict for some $h\in \{h_{1},\ldots ,h_{k}\}$. Note that for any $h\notin \{h_{1},\ldots ,h_{k}\}$, $b^{i}(h,\eta )=1$.

Hence, if $u_{r_{i}}(\{h_{1},\ldots ,h_{k}\})<u_{r_{i}}(S')$, this means if all agents $r_{1},\ldots ,r_{k-1}$ are being assortative (so $b^{k}(h,\eta )=1=b^{i}(h,\eta )$ for $h\in S'{\setminus } \{h_{1},\ldots ,h_{k}\}$), $u_{r_{k}}(\{h_{1},\ldots ,h_{k}\})<u_{r_{k}}(S')$. That is, if it is not beneficial for $r_{i}$ to be assortative, it would not be beneficial for $r_{k}$ to be assortative if $r_{1},\ldots ,r_{k-1}$ are assortative.

Note that, as all these players have a strictly dominant strategy, this is a unique equilibrium for this game. $\square$

### Interviewing equilibria when preferences are drawn from the Mallows model

While Theorems 1 and 2 hold for general preferences, we are interested in understanding the impact that the underlying preference model has on the strategic choices of the residents. To this end, we investigate the strategic behaviour that arises when residents’ preferences are drawn from the $\phi$-Mallows model (Mallows 1957), a probabilistic ranking model that is standardly used for modelling preferences and has been used in previous investigations of preference elicitation schemes for stable matching problems (Drummond and Boutilier 2013, 2014; Brilliantova and Hosseini 2022; Freeman et al 2021). Its particular relevance in our setting is that residents often have a vague ranking of hospitals, based on a common list (e.g., US News Ranking of hospitals)—the Mallows reference ranking—but in practice, their personal preferences may be a noisy variant of it.

#### The Mallows model

The $\phi$-Mallows model (or just Mallows model Mallows (1957)), $D ^{\phi ,\sigma }$, is a distance-based probabilistic ranking model, characterized by a reference ranking $\sigma$, and a dispersion parameter $\phi \in (0,1]$. Given the parameters, $\sigma$ and $\phi$, the probability of any given ranking $\eta$ is:

$$\begin{aligned} P(\eta | D ^{\phi ,\sigma })&= \frac{\phi ^{d(\eta ,\sigma )}}{Z} \end{aligned}$$

where $d(\eta , \sigma )$ is the Kendall-$\tau$ distance metric that counts the number of pairwise disagreements between the two rankings, $\eta$ and $\phi$, and *Z* is a normalization factor; $Z = \sum _{\eta \in A_\succ } \phi ^{d(\eta ,\sigma )} = (1)(1+\phi )(1+\phi +\phi ^2)\ldots (1+\cdots +\phi ^{|A|-1})$ (Lu and Boutilier 2011). The parameter $\phi$ controls the likelihood of drawing a ranking that is significantly different from the reference ranking, $\sigma$. As $\phi \rightarrow 0$, the probability of drawing the reference ranking approaches 1.0, while as $\phi \rightarrow 1$, the Mallows distribution is equivalent to drawing a ranking from the uniform distribution.

One interpretation of the Mallows model has rankings being generated by inserting alternatives into a ranking, where the insertion point is a function of $\phi$. Because of this, when comparing only a small subset of alternatives in the ranking, the probability that any two of the alternatives of interest are in a specific order may not depend on the total number of alternatives. Furthermore, it is possible to determine the probability that any given alternative will be inserted in a particular position in a ranking simply by computing the probability it will be inserted in that position after all other alternatives have been ranked. We will use these properties in our analysis and so state them here and include the proofs in Appendix A for completeness.

##### Lemma 1

Given some Mallows model $D ^{\phi ,\sigma }$ with a fixed dispersion parameter $\phi$ and reference ranking $\sigma$ ordering *n* agents, in which $a_i~\succ ~a_j$ ($1\le i,j\le n$), the probability that a ranking $\eta$ is drawn from $D ^{\phi ,\sigma }$ such that $a_i \succ _{\eta } a_j$ is equal to drawing from some distribution $D ^{\phi ,\sigma '}$ where $\sigma$ is a suffix or prefix of $\sigma '$ (that is, there is $\sigma$, an ordering of *n* agents, and $\sigma '$, an ordering of $n'$ agents ($n'>n$), and $\sigma '$ can be divided into $\sigma$, an ordering of the first/last *n* agents, and an ordering of the last/first $n'-n$ agents).

##### Corollary 1

Given any reference ranking $\sigma$ and two adjacent alternatives in $\sigma$: $a_i,a_{i+1}$,

$$P(a_i \succ a_{i+1} | D^{\phi ,\sigma }) = \frac{1}{1+\phi }.$$

We extend Corollary 1 to include three consecutive items.

##### Corollary 2

Given any reference ranking $\sigma$ and alternatives $a_{i},a_{i+1},a_{i+2}$ and some $\eta ~\in ~\{a_i,a_{i+1},a_{i+2}\}_\succ$, the probability that some ranking $\beta$ is drawn from $D ^{\phi ,\sigma }$ that is consistent with $\eta$ is:

$$P(\beta | D ^{\phi ,\sigma }) = \frac{\phi ^{d(\eta ,a_i \succ a_{i+1} \succ a_{i+2})}}{(1+\phi )(1+\phi +\phi ^2)}$$

It is useful to know the probability that any one alternative will be in any particular position in a rank ordered list. We show that this is effectively equivalent to ordering all other alternatives, and then calculating the probability that we can put the alternative in question in its desired slot.

##### Lemma 2

The probability that $a_{1}$ will be ranked in place *j* is $\frac{\phi ^{j-1}}{1+\phi +\cdots +\phi ^{n-1}}$. Furthermore, the probability that $a_{n}$ will be ranked in place *j* is $\frac{\phi ^{n-j}}{1+\phi +\cdots +\phi ^{n-1}}$. Similarly, the probability $a_{j}$ will be ranked in first place is $\frac{\phi ^{j-1}}{1+\phi +\cdots +\phi ^{n-1}}$.

It is possible to bound the probability that any two alternatives will be “out of order” in any given ranking;

##### Lemma 3

Let $\eta \in D ^{\phi ,\sigma }$ be such that $a_{j}\succ _{\eta }a_{i}$ for some $i<j$, then $P(\eta )<\frac{\phi ^{j-i}}{Z}$.

Finally, we include an observation that follows from the definition of the Mallows’ model:

##### Observation 1

If $|j-i|>|j-i'|$, probability $a_{i}$ is in place *j* is smaller than probability $a_{i'}$ is in place *j*. Similarly, probability $a_{j}$ is in place *i* is smaller than probability $a_{j}$ is in place $i'$.

#### Equilibrium analysis

We now study the equilibria that arise in the interviewing game when residents’ preferences are drawn from some underlying $\phi$-Mallows model. This allows us to control and, thus, better understand, how diversity of residents’ preferences influences the structure of the underlying interviewing equilibrium. For ease of notation, let $\Psi = \langle k,\phi ,v\rangle$ be an instance of an **ILQ** game with interview quota *k*, a Mallows model with dispersion parameter $\phi$, and a scoring function *v*.

We start by considering the class of games where $\phi =0.0$, namely $\Psi =\langle k,0.0,v\rangle$. Recall that as $\phi \rightarrow 0$, the probability of drawing the reference ranking $\sigma$ goes to 1. This means that all residents have common preferences, namely the reference ranking which we define as $\sigma$ such that $h_i\succ h_{i+1}$ for all $1\le i<n$. It is straightforward to see that any strategy profile such that each resident $r_i$ interviews with hospital $h_i$ forms an equilibrium. Thus, trivially, assortative interviewing is an equilibrium as well.

We now consider the general case, $\Psi =\langle k,\phi ,v\rangle$, where no restrictions are placed on any of the three parameters. We observe that if resident $r_k$ can not improve its expected utility by interviewing with hospital $h_{k+1}$ instead of any of the hospitals in $\{h_1,\ldots , h_k\}$, then in general the best thing resident $r_k$ can do is set its interview set to be $I(r_k)=\{h_{1},\ldots , h_k\}$. We formalize this in Lemma 4 and defer the proof to Appendix B. Note that this result greatly simplifies the equilibrium analysis going forward: we need only consider *k* possible interviewing sets, instead of $$\left( {\begin{array}{c}n\\ k\end{array}}\right) $$ to determine if assortative interviewing is the best strategy for $r_k$.

##### Lemma 4

Given **ILQ** game $\Psi = \langle k,\phi ,v \rangle$, if resident $r_k$’s expected payoff from interviewing with hospitals $\{h_1,\ldots ,h_k\}$ (when residents $r_{1},\ldots ,r_{k-1}$ have interviewed with them as well) is higher than their expected payoff from interviewing with hospitals $\{h_1,\ldots ,h_{k+1}\} {\setminus } \{h_j\}$ for all $j \in \{h_1,\ldots ,h_k\}$, then resident $r_k$’s best response is to interview with $\{h_1,\ldots ,h_k\}$ (i.e., assortatively).

We now provide a necessary and sufficient condition for assortative interviewing to hold for **ILQ** game $\Psi = \langle k,\phi ,v \rangle$. Let $P(h_i)$ denote the probability that hospital $h_i$ is available for resident $r_k$ (i.e., residents $r_1,\ldots ,r_{k-1}$ are all matched to different alternatives).

##### Lemma 5

Given **ILQ** game $\Psi = \langle k,\phi ,v\rangle$, if residents $r_1,\ldots ,r_{k-1}$ all interview assortatively (i.e., with hospital set $S = \{h_1,\ldots ,h_{k}\}$), then assortative interviewing is a best response for resident $r_k$ if and only if the following inequality is satisfied for all $h_j \in \{h_1,\ldots ,h_k\}$ when $S^\prime = S {\setminus } \{h_j\} \cup \{h_{k+1}\}$:

$$\begin{aligned} P(h_j )\mathbb {E}(v(h_j) | D ^{\phi ,\sigma })&\ge \quad P(h_j ) \mathbb {E}(v(h_{k+1}) | D ^{\phi ,\sigma })\\&+ \sum _{\eta \in H_\succ } P(\eta | D ^{\phi ,\sigma }) \cdot \big [\sum _{h_i \in S^\prime } P(h_i )\mathbb {1}_{h_{k+1}\succ _\eta h_i}v(h_{k+1},\eta ) \big ] \nonumber \end{aligned}$$

where

$$ \mathbb {1}_{h_i \succ _\eta h_j} = {\left\{ \begin{array}{ll} 1,& {if }\, h_i \succ _\eta h_j \\ 0, & {otherwise}\, \end{array}\right. } $$

We now present our key result for this section. We provide a necessary and sufficient condition for assortative interviewing to form an equilibrium for a given **ILQ** game, $\Psi =\langle k,\phi , v\rangle$. Furthermore, this condition can be checked efficiently since it only involves checking *k* possible interview sets for a single resident, $r_k$.

##### Theorem 3

Given **ILQ** game $\Psi = \langle k,\phi ,v \rangle$, then satisfying the inequality found in Lemma 5 for all $h_j \in \{h_1,\ldots ,h_k\}$ is both sufficient and necessary to show that all residents interviewing assortatively form an equilibrium for this game.

##### Proof

For the first *k* residents, this follows directly from combining Theorem 2 and Lemma 5. The theorem would be correct if we could apply this proposition and lemma iteratively, one group of *k* hospitals and residents at a time. Thanks to the Mallows distribution’s properties, we can: If $r_{k}$’s best response was assortative, we know that all the residents $r_{1},\dots ,r_{k}$ interviewed assortatively, thus all hospitals $h_{1},\ldots , h_{k}$ are taken. This means that the same equations that told us that $r_{k}$’s best response (to $r_{1},\ldots ,r_{k-1}$) was assortative tell us that $r_{2k}$’s best response (to $r_{k+1},\ldots ,r_{2k-1}$) is assortative: Since a switch between $h_{1}$ and $h_{2}$ has the same probability as switching between $h_{k+1}$ and $h_{k+2}$, if Theorem 2 and Lemma 5 can be applied once on hospitals and residents $1,\dots , k$, they can be applied again for $k+1,\ldots ,2k$, as all equations remain the same, due to the practical “disappearance” of the hospitals $h_{1},\ldots ,h_{k}$ for agents $r_{k+1},\ldots ,r_{2k}$ (thus their order can be ignored). Now that we have shown that the first two groups of *k* residents interview assortatively, we can use the same argument iteratively for the next *k* residents, and so on. $\square$

To conclude this section we note that while we focussed on assortative equilibria since they are elegant and simplifies the problem of determining equilibrium strategies for the residents, other equilibria may also exist. For example, consider the special case where $\Psi =\langle k, 1.0, v \rangle$. When $\phi =1.0$ the resulting Mallows distribution is uniform. As first noted by Lee and Schwarz (2017) under a different model, when residents and hospitals are divided into *n*/*k* subsets and matched inside these subsets, this also forms an equilibrium.

## Assortativity, utility function structure, and quotas

We now focus our attention on understanding the interplay between the number of interviews residents may conduct and the structure of the underlying utility functions. We continue to be interested in characterizing the conditions in which “natural” or assortative interviewing equilibria exist.

To ground the work we continue to assume that residents’ preferences are drawn from some underlying ranking distribution generated by the $\phi$-Mallows model, and then we instantiate the residents’ utility functions in three different ways, drawing inspiration from both the social choice literature (Brandt et al 2016; Loewenstein et al 1989; Messick and Sentis 1985) and the matching literature (e.g. Coles and Shorrer 2014; Calsamiglia et al 2020). Let $h_i$ be the $i^{th}$ ranked hospital in a resident’s ranking $\eta$.

**Plurality-based:** A utility function is *plurality-based* if
  $$\begin{aligned} v(h_i) = {\left\{ \begin{array}{ll} 1, & {\text {if }}\,i=1 \\ 0, & i>1 \end{array}\right. } \end{aligned}$$
**Borda-based:** A utility function is *Borda-based* if for any $h_i$,
  $$\begin{aligned} v(h_i) = n-i+1 \end{aligned}$$
  where *n* is the number of alternatives (hospitals) in the market. This is equivalent, in a sense, to the expected rank, though the values are inverted—the most preferred choice has a maximal Borda score, but the expected rank value is minimal (1).
**Exponential:** A utility function is exponential if
  $$\begin{aligned} v(h_i)=\left( \frac{\epsilon }{2}\right) ^{i-1}, {\text {for }}\, 0<\epsilon <1. \end{aligned}$$

These three functions capture a wide range of residents’ preferences. If best modelled using plurality-based utility functions, residents care only about being matched to their top choice. Borda-based, on the other hand, provides a linear utility function that decreases as a resident is matched to a less preferred hospital. The class of exponential utility functions forms a bridge between plurality and Borda.

Our first result identifies a condition under which residents with plurality-based utility functions will interview assortatively in equilibrium. The proofs are provided in Appendix D.

### Lemma 6

Given **ILQ**game $\Psi =\langle k, \phi , v\rangle$ where *v* is the plurality-based utility function, a necessary and sufficient condition for assortative interviewing to be an equilibrium is

$$\begin{aligned} P(h_j)\ge \phi ^{k-j+1} \end{aligned}$$

where $P(h_j)$ is the probability that hospital $h_j$ is available for resident $r_k$.

There is a strong relationship between the conditions under which assortative interviewing is an equilibrium when residents have plurality-based utility functions and when they have exponential utility functions.

### Lemma 7

Given **ILQ**game $\Psi =\langle k, \phi , v\rangle$, if

$$\begin{aligned} P(h_j)\ge \phi ^{k-j+1} \end{aligned}$$

when *v* are plurality-based utility functions, then there exists exponential utility functions that also result in assortative interviewing being in equilibria.

One can immediately develop some intuition from these Lemmas by considering the extreme values for the $\phi$-parameter. For example, if $\phi =1.0$, then the distribution from which residents’ preferences are drawn is uniform.^5^ In this case, assortative interviewing will only be supported in equilibrium if resident $r_k$ is certain to be matched with $h_1$. This is clearly very strong and unlikely to hold in many real-world settings. On the other hand, if $\phi$ is close to zero, meaning that residents’ true rankings over hospitals are likely to be similar to each other, then assortative interviewing is supported as long as there is some (possibly fairly small) chance that $h_1$ will be available to be matched to $r_k$. We will leverage Lemmas 6 and 7 in the rest of this section to gain a clearer picture of the characteristics of assortative equilibria.

### Assortative equilibria when $k=2$

If residents are only allowed to interview with two hospitals, then assortative interviewing forms an equilibrium under certain conditions. In particular, the existence of assortative interviewing depends on both the structure of the residents’ utility functions and the dispersion parameter, $\phi$, of the underlying Mallows model.

#### Theorem 4

Given **ILQ**game $\Psi =\langle k, \phi , v\rangle$ with $k=2$ and *v* being plurality-based utility functions, assortative interviewing forms an equilibrium when $0 < \phi \le 0.6180$.

A direct consequence of Theorem 4 and Lemma 7 is that for exponential scoring functions, when $0< \phi < 0.6180$, there exists an $\varepsilon$ such that if residents’ scoring function is an exponential function dominated by $(\frac{\varepsilon }{2})^{(i-1)}$ with $\varepsilon > 0$, assortative interviewing is an equilibrium for that $\phi$.

We are also able to show a similar result when the utility functions of residents are Borda-based, though assortative interviewing is in equilibrium for a significantly smaller range of $\phi$, meaning that the preferences of the residents are much less diverse. This illustrates the strong connection and interplay between the structure of the utility functions of the residents, the underlying preference distribution, and the number of interviews residents may participate in.

#### Theorem 5

Given **ILQ**game $\Psi =\langle k, \phi , v\rangle$ with $k=2$ and *v* being Borda-based utility functions, assortative interviewing forms an equilibrium when $0 < \phi \le 0.265074$.

### Assortative equilibria when $k=3$

Interestingly, when residents can interview with up to three hospitals, assortative interviewing continues to be an equilibrium for plurality-based and exponential utility functions but is no longer an equilibrium if residents have Borda-based utility functions. We begin with the negative result for Borda-based utility functions.

#### Theorem 6

Given **ILQ**game $\Psi =\langle k, \phi , v\rangle$ with $k=3$ and *v* being Borda-based utility functions, then assortative interviewing may not form an equilibrium or any $0<\phi \le 1$.

Alternatively, for plurality and exponential-based utility functions, assortative interviewing still forms an equilibrium for certain ranges of $\phi$ in the Mallows model. We observe, however, that the range of $\phi$ is smaller than in the case where $k=2$, indicating again the sensitivity of residents’ strategic decisions on all aspects of the **ILQ**game.

#### Theorem 7

Given **ILQ**game $\Psi =\langle k, \phi , v\rangle$ with $k=3$ and *v* being plurality-based utility functions, assortative interviewing forms an equilibrium when $0 < \phi \le 0.4655$.

The existence of assortative interviewing, when *v* are exponential-based, is an immediate consequence of Theorem 7 and Lemma 7.

### Assortative equilibria when $k\ge 4$

We finally consider the setting where residents can interview with more than three hospitals. Unfortunately, our results are negative; we show there are settings, characterized by *k* and $\phi$, such that assortative interviewing is not an equilibrium, irrespective of the underlying utility function. We begin by showing that when there is a setting for which there is no assortative equilibria for plurality, then there is no scoring function with assortative equilibria. We use this result to show that, for sufficiently small dispersion parameter $\phi$ and for $k > 3$ interviews, assortative interviewing cannot be an equilibrium under any scoring function. We then provide a specific counterexample for *all* $\phi$ when $k = 4$ for plurality, implying there is no assortative equilibrium for any scoring function. This suggests that, for a wide category of resident valuation functions under a Mallows distribution, contrary to some real-world behaviour, assortative interviewing is not an equilibrium.

#### Theorem 8

Given **ILQ**game $\Psi = \langle k,\phi ,v\rangle$ with $k\ge 4$ and *v* being plurality-based, if hospital $h_1$ causes the condition in Lemma 5 to be falsified (i.e., $\{h_2,\ldots ,$ $h_{k+1}\}$ has a better expected payoff than $\{h_1,\ldots ,h_k\}$), then for $k\ge 4$ and $\phi$, assortative interviewing is not an equilibrium for any valuation function.

Intuitively, there is a tradeoff between the likelihood that a hospital will be available for resident $r_k$ by the time it is their turn to be matched and the expected value of that hospital. Both are strongly tied to the dispersion parameter $\phi$ of the Mallows model: as the dispersion parameter approaches 1.0, the difference in the expected value of any given hospital goes to 0. As the dispersion parameter approaches 0.0, the expected value of any hospital $h_i$ goes to the value of its slot in expectation, $v(s_i)$. However, the likelihood it is taken by some higher ranked $r_j$ (i.e., with $j < i$) also approaches 1. The following theorem addresses the latter case: for sufficiently small dispersion, even though the expected value of a hospital is high, the likelihood it will be available is so low that residents are disincentivized from choosing to interview with it.

#### Theorem 9

Given **ILQ**game $\Psi = \langle k,\phi ,v\rangle$ with $k>4$, there exists $0<\varepsilon <1$ such that for any scoring function *v* no assortative interviewing forms an equilibrium for dispersion parameter $0< \phi <\varepsilon$.

We now show that for $k = 4$, assortative interviewing is not an equilibrium for *any* $\phi < 1$ and any scoring rule. We then continue to show that for $k > 4$ and $\phi$ sufficiently small, assortative interviewing is not an equilibrium.

#### Theorem 10

Given **ILQ**game $\Psi = \langle k,\phi ,v\rangle$ with $k = 4$ and any scoring function v, assortative interviewing is not an equilibrium for any dispersion parameter $0 \!<\! \phi \!< \!1$.

It seems unlikely that for $k > 4$, assortative interviewing is an equilibrium. Intuitively, if it is an equilibrium it should be for low $\phi$: this is when the expected value of hospital $h_i$ is very close to $v(s_i)$. However, this is also when residents $r_1,\ldots ,r_{k-1}$ are all most likely to be matched with hospitals $h_1,\ldots ,h_{k-1}$. We leave open the possibility that there may exist some $\delta$ such that when $0< \varepsilon< \phi < \delta \le 1$, assortative interviewing is an equilibrium for plurality.

## Beyond assortative interviewing

The results in the previous sections are mixed. While we believe that assortative interviewing is an interesting phenomenon and is “natural” as it provides an intuitive strategy for residents, we have also shown that such equilibria are only guaranteed to exist when residents have a limited number of interviewing options.

This inspires us to do two things. First, we observe that our definition of assortative interviewing is strong. Thus, we explore the ramifications of weakening the definition. As we will show, interestingly, our weaker definition does not help and instead can add further complications to the problem. This motivates us to expand the class of what we consider “natural” outcomes and initiate an investigation into the class of *reach and safety* strategies.

### The weakness of weak assortative strategies

Our definition of assortative interviewing was very strong. Definition 1 imposed two key restrictions. First, it assumed that each resident, with an interview budget of size *k*, interviews with *k* consecutive hospitals (according to the reference ranking). Second, the definition assumed that the top *k* residents interviewed with the top *k* hospitals, the following *k* residents (ranked from $k+1$ to 2*k*) interviewed with the next *k* hospitals, etc. We consider two relaxations of this definition: *pseudo-strong assortative* and *weak assortative* interviewing.

#### Definition 2

Given **ILQ**game with quota $k>0$, an interviewing strategy profile is *pseudo-strong assortative* if given $j = 0,1,2, \ldots , \frac{n}{k}-1$, for each group of *k* hospitals such that $H_j=\{h_{1+kj},h_{2+kj},\ldots , h_{k(j+1)}\}$, there exists a subset of residents, $R_j\subset R$, $|R_j|=k$ such that all residents in $R_j$ interview with $H_j$.^6^

Note that this definition relaxes the assumption that consecutive residents interview with the same hospitals. For example, if there was an outcome such that resident $r_{1}, r_{3}$ and $r_{5}$ interview at $h_{1},h_{2}$ and $h_{3}$, while residents $r_{2}, r_{4}$ and $r_{6}$ interview at hospitals $h_{4}, h_{5}$ and $h_{6}$, this would be pseudo-strong but not strongly assortative.

#### Definition 3

Given **ILQ**game with quota $k>0$, we say that an interviewing strategy profile is *weakly assortative* iff for all $r_i\in R$, $I(r_i)=\{h_j,h_{j+1},\ldots ,h_{j+k-1}\}$ for some *j*.

Weak assortative interviewing has that each resident selects *k* consecutive hospitals to interview and places no other restrictions on the residents’ strategies. That is, it is conceivable that almost every resident is targeting a different part of the hospital list.

Strong and pseudo-strong strategies result in a similar structure of resident and hospital interviews: hospitals are divided into consecutive sets (the top-*k* hospitals, the 2nd-*k* hospitals, etc.), and each resident interviews in one of those sets. Since a resident only interviews with one of those sets (e.g., a resident cannot interview in some of the top-*k* and some of the 2nd-*k*), such a structure may happen when the difference between the expected value of each hospital is so small that the mere interview of a resident in a hospital (which decreases the probability of another resident getting it) reduces the expected value in a way that another agent prefers to avoid it completely. This can happen when $\phi$ is very close to 1, in which case all hospitals are almost equivalent to resident preferences. It can also occur when the valuation function is such that the value of each hospital is very close, regardless of their ranking.

Since residents do not interview with different sets of hospitals (top-*k*, 2nd-*k*, etc.), this also means that if $r_{1}$ and $r_{2}$ interview in the same hospitals in equilibrium, this indicates $r_{2}$ sees some value in these hospitals, even if $r_{1}$ interviews there, which means that $r_{3}$ would consider these hospitals as well (assuming $k>2$, of course), so while $r_{3}$ may gradually change the interview set, they will not completely avoid $r_{1}$ and $r_{2}$’s hospitals (resulting in an equilibrium that is not strong or pseudo-strong).

The above argument, however, does not answer what happens when we consider weakly assortative interviewing that is neither strong nor pseudo-strong. Surprisingly, weak assortative interviewing turns out to be more problematic than strong (or pseudo-strong) assortative interviewing.

#### Theorem 11

Given **ILQ**game $\Psi =\langle k, \phi , v\rangle$ where all residents employ a weakly assortative interviewing strategy (that is, at least one resident is not strong or pseudo-strong). Then there is a non-zero probability that some resident will be unmatched.

Note that such a situation cannot happen with strongly (or pseudo-strong) assortative strategies—sets of *k* residents all interview at the same *k* hospitals, meaning they are guaranteed to be matched.

While Theorem 11 is an inherently negative result, it is not the only negative aspect that arises when one considers weakening strong assortativity.

#### Theorem 12

Given **ILQ**game $\Phi =\langle k,\psi , v\rangle$ with $k=2$, $\phi <1$, and $|R|=|H|=4$, then there does not exist a Nash equilibrium in which residents have weak-assortative strategies.

**Fig. 1:**
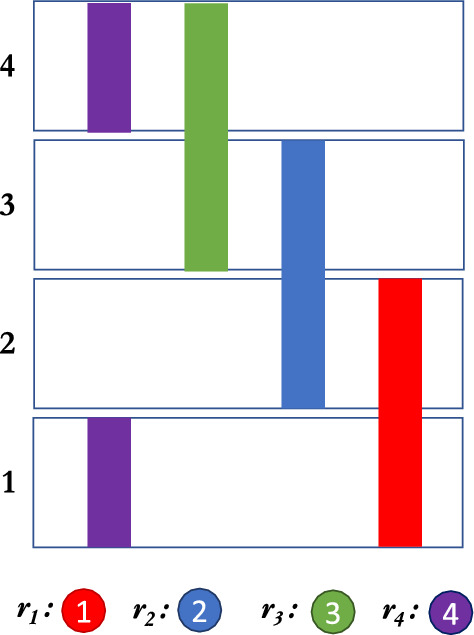
Interviewing sets when $|R|=4$, $k=2$, and we require weak assortative strategies.

The proof of Theorem 12 (found in the appendix) illustrates a cascading effect where residents (particularly the bottom-ranked resident) have incentives to break the sequential structure of hospitals when selecting their interview sets. This is illustrated in Fig. 1. This is further exacerbated as the number of residents (|*R*|) increases. With a larger *k*, a similar process would occur, and as also intuited in Theorem 11, creating more than *k* residents interviewing at the same hospital means the probability of a resident being left without a hospital grows. While we hypothesize that for $k > 2$ and $n > k$, there is no Nash equilibrium at all with only weakly assortative strategies (in cases without tie-breaking), it is clear that if there is a sufficiently negative cost to being left without a hospital (as there is in the real world), weakly assortative interviewing cannot happen, as weakly assortative strategies result in hospitals with over *k* interviewees (and thus residents without hospitals). These residents would instead seek to reduce this probability by interviewing at the hospitals with highest availability probability, which means they would not interview in a hospital with more than *k* interviewees.

### Reach and safety strategies for a small interviewing quota

While the example shown in Fig. 1 lacked the assortativity structure we have been interested in, it still illustrated an interesting phenomenon, *reach-and-safety* strategic behaviour. We investigate such behaviour empirically and relate the emergence of such behaviour to the underlying preference models of the residents. We focus on small **ILQ**games as we concentrate on exact computation of the underlying equilibrium, but we hypothesize that our findings generalize to larger settings.

Consider the case for $k=2$ interviews where (for the Borda scoring rule) we only guarantee assortative interviewing for some sufficiently small dispersion parameter $\phi$. To gain better insight into the strategic behaviour of the residents as a function of $\phi$, we calculated the exact values of $\phi$ where the interviewing equilibria changes in small markets. In doing so, we see that the structure of the interviewing equilibria contain both “reach” and “safety” schools, where participants diversify their interviewing portfolio to get both the benefit of a desirable, unlikely option, and a likely, but less desirable option.

**Fig. 2:**
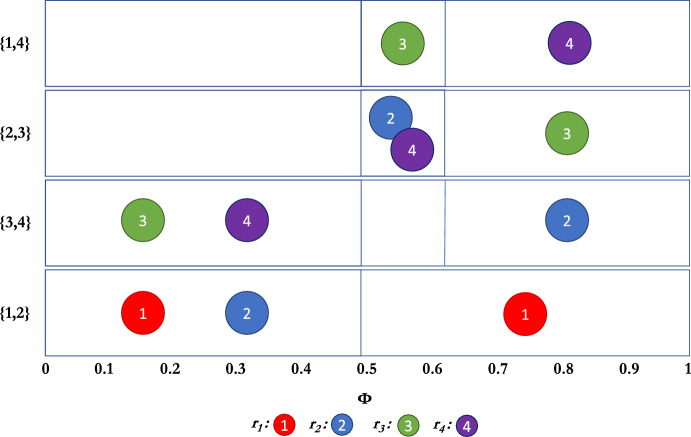
Interviewing sets of residents as a function of $\phi$ when using the Borda scoring function, with 4 participants, and interview set size of 2.

Figure 2 depicts a market with 4 hospitals, 4 residents, and 2 interviews ($n = 4$, $k = 2$). The figure shows what sets are being chosen by the different residents for any dispersion $\phi$. As $\phi$ increases, we explicitly see the trade-off between a safer choice, and a better expected-payoff value for individual alternatives. For small $\phi$, as the theoretical results showed, assortative interviewing is optimal, and $r_{2}$ chooses $\{h_1,h_2\}$, while $r_{3}$ and $r_{4}$ choose $\{h_{3},h_{4}\}$. Interestingly, for $\phi \in [0.5,0.62]$, $r_2$’s best option is to split the difference, and interview with one hospital ($h_3$) they are guaranteed to get and one hospital ($h_2$) that will be available with sufficiently high probability, and has a higher expected value. This choice available to $r_2$ further results in some of the “reach” vs. safe behaviour we see in college admissions markets; namely, $r_3$’s best response now is to interview with $h_1,h_4$ (i.e., a “reach” choice, and a “safe” bet), while $r_{4}$, being left without any truly “safe” option, aims slightly higher than its rank. As $\phi$ grows and approaches 1, any ordering of hospitals is as likely as another, making $r_{2}$’s choice $\{h_{3},h_{4}\}$, which are as likely as any to be highly ranked, and are available. The desire to avoid interviewing hospitals that are already chosen by many other residents also drives $r_{3}$ and $r_{4}$ to $\{h_{2},h_{3}\}$ and $\{h_{1},h_{4}\}$, respectively; that is, they both want to avoid competing with $r_1$ and $r_2$.

**Fig. 3:**
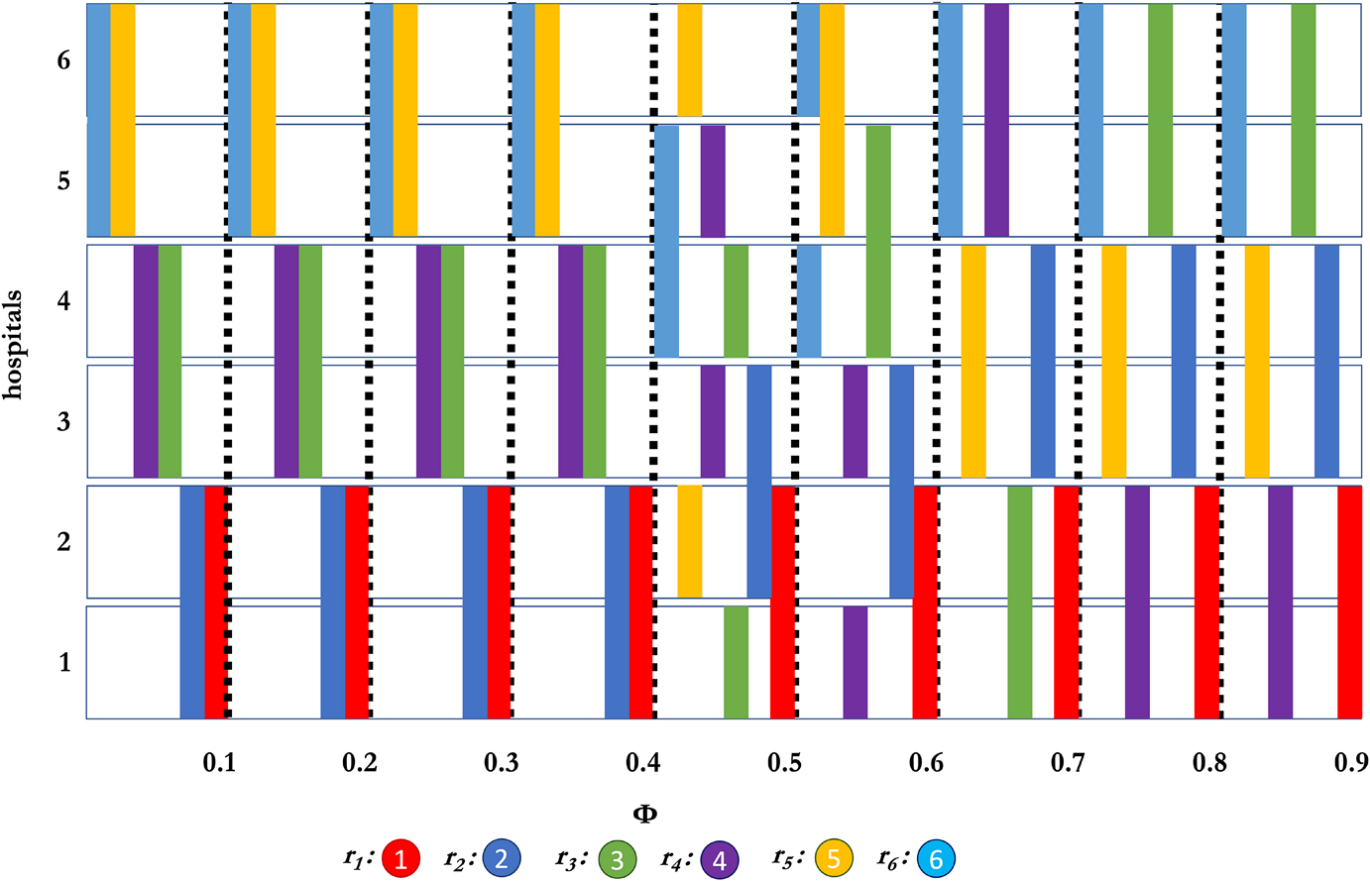
Interviewing sets of residents as a function of $\phi$ when using the Borda scoring function, with 6 participants, and interview set size of 2.

**Fig. 4:**
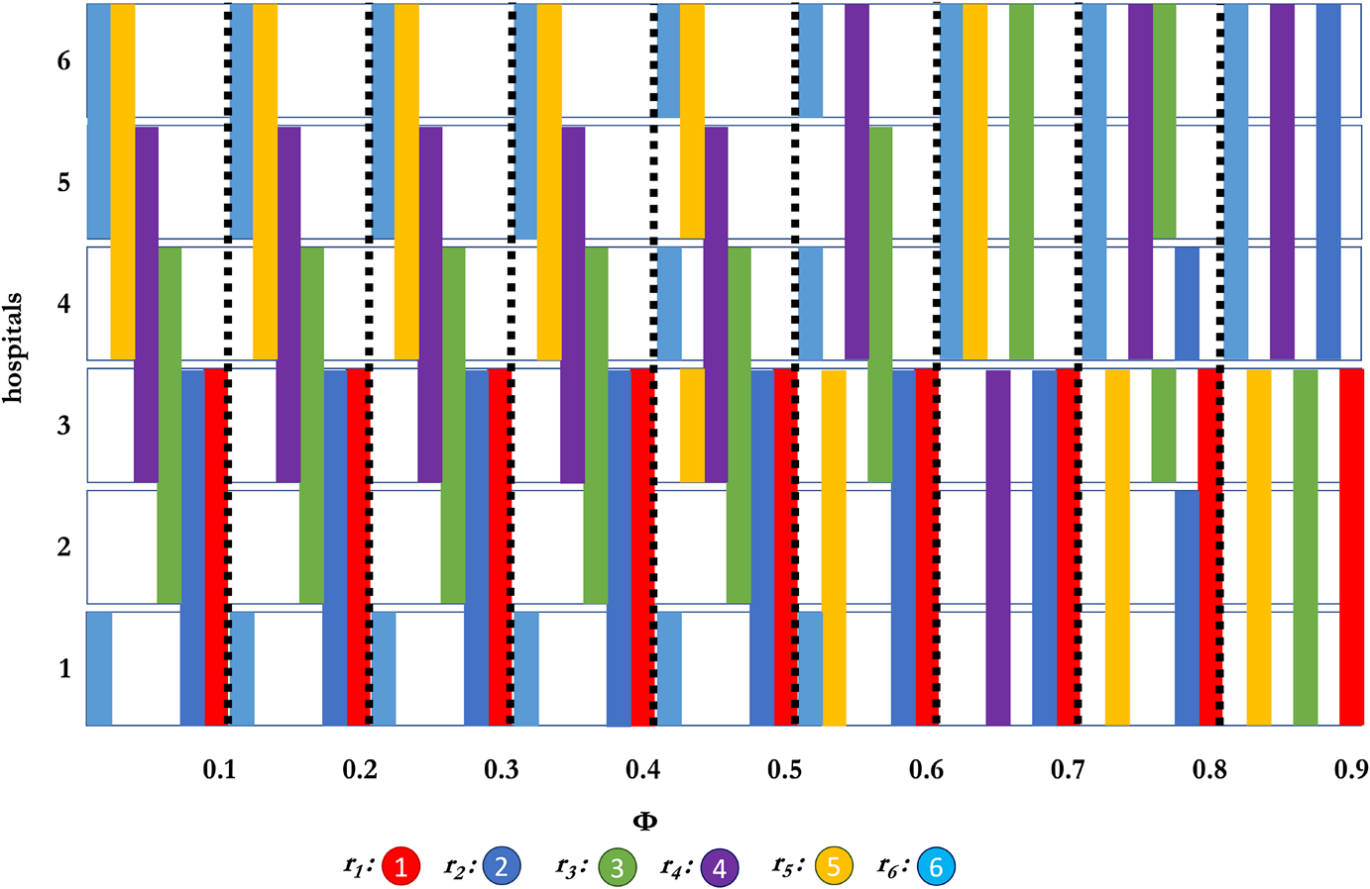
Interviewing sets of residents as a function of $\phi$ when using the Borda scoring function, with 6 participants, and interview set size of 3.

**Fig. 5:**
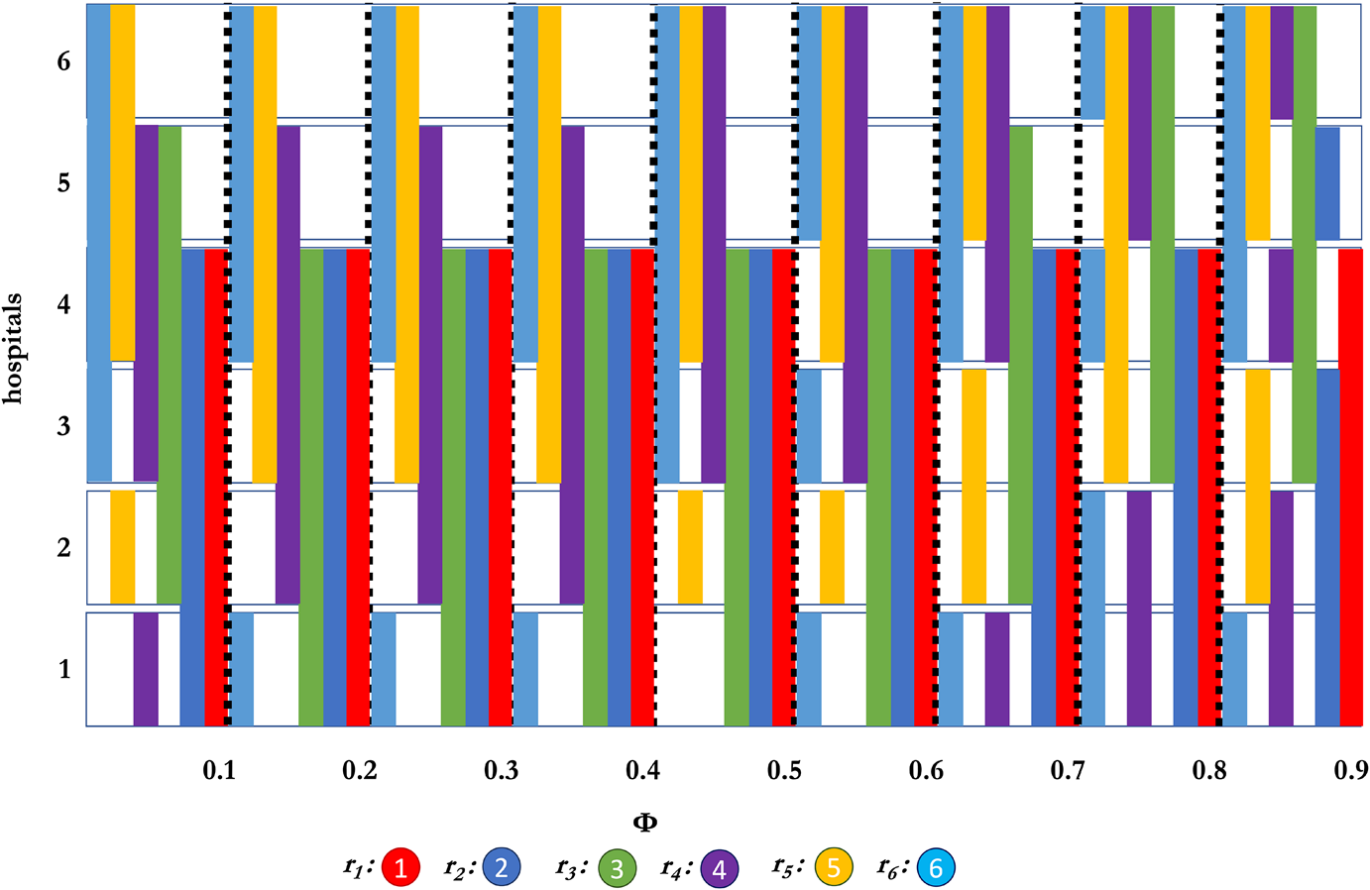
Interviewing sets of residents as a function of $\phi$ when using the Borda scoring function, with 6 participants, and interview set size of 4.

We expand on these results and now consider the case of $n=6$ residents with $k=2, 3$ and 4 interviews per resident. Here we see in Figs. 3, 4, and 5, similar equilibrium strategies as for the $n=4,k=2$ case. For $k=2$, and $\phi \le 0.4$, we again see that assortative interviewing is an equilibrium. When $\phi = 0.5$, we observe that the second resident departs from strict assortative interviewing in favour of a weak version of assortative interviewing and this, in turn, affects the other players, as, for example, the third resident applies what is a safety move (hospital 4, which is theirs if they want it) with a reach move (hospital 1, the top choice). Of some interest, for $\phi \ge 0.7$, all residents except $r_{1}$ use a weak assortative strategy, that is, they interview in sets of hospitals which are adjacent in rank, rather than splitting their interviews between radically different ranked hospitals.

Turning to $k = 3$ interviews per resident, we see as Theorem 6 claimed, that the third resident does not interview assortatively. While residents 3, 4, and 5 are mostly weakly assortative (except the third resident and $\phi = 0.8$, where it tries a small reach choice), the sixth resident goes consistently for a reach and safety strategy, as it interviews in the top hospital as well. The resident’s behaviour only changes when $\phi$ is large enough ($\phi >0.6$) when the chance of the true ranking being different from the ground truth is much higher. Of interest, when $\phi = 0.9$, the second resident chooses hospitals 4, 5, 6 (even knowing that at least two of the hospitals in {1,2,3} will be available. But when $\phi$ is sufficiently close to 1, the distribution is approaching the uniform distribution so that this resident might as well choose hospital 4, 5, 6 as they might very well be as desirable as 1, 2, 3 where the residents’ top choices might be taken.

Finally, for $k = 4$, we see that resident 1 (as we know must happen) interviews assortatively for all settings of $\phi$ while other residents are much more willing to experiment. Not included in Fig. 5 are further results, showing that even for some very small $\phi$ ($0.1>\phi \ge 10^{-20}$), there are residents which are not even weakly assortative. We hypothesize that this “reach” and “safety” behaviour is present in markets with larger interviewing quotas.

## Conclusions and future directions

We investigated equilibria in **ILQ**games, inspired by the matching of medical residents to hospital programs. A key feature of this game is that residents must interview with hospitals to discover their true preferences, but are limited in the number of interviews they may conduct. This introduces a new level of complication as residents need to carefully consider how to optimize their interviewing strategies given the interviews choices of other residents. While we showed the existence of a pure-strategy Nash equilibria for this game, we where particularly interested in understanding under what circumstances assortative interviewing forms an equilibrium, as such strategies are “easy” for residents to execute, result in stable outcomes where everyone is matched, and which earlier work had suggested might exist (Lee 2017).

We summarize our findings into a few key take-aways that may provide useful guidelines for market designers:

- Assortative interviewing is supported in equilibrium, but its existence depends critically on how correlated residents’ preferences are, the limit on the number of interviews, and the structure on the underlying value functions for alternatives.
- If the underlying value function is Borda-based, then assortative interviewing only forms an equilibrium when preferences of residents are closely correlated (as measured by the $\phi$ parameter in the underlying $\phi -$Mallows model). If the underlying value function is plurality-based or exponential then assortative interviewing is more broadly supported.
- Limiting the number of interviews residents can undertake is critical if assortative interviewing it to be supported. If residents are allowed to interview with 4 or more hospitals then assortativity might not be supported in equilibrium.
- Relaxing the definition of assortativity (to weak-assortativity) does not help.

There are many research questions raised by our results, to which at least some of out technical results and techniques may also contribute. Most concretely, we hypothesize Theorem 9 could be replaced by extending Theorem 10 for all $k\ge 4$. Second, while we believe that the space of scoring functions used in this paper was broad in its scope, we always assumed that residents’ underlying ranked preferences were drawn from a distribution generated by the $\phi$-Mallows model. While the $\phi$-Mallows model is standard in the literature, it is possible that other preference distributions (e.g., Plackett-Luce) may better support assortative interviewing. Second, the analysis relies on the assumption that one side of the market maintained a master list. While master-lists do occur in real-world matching markets, lifting this assumption will obviously generalize the setting, and may invalidate our results. More specifically, the removal of the master-list assumption would complicate the analysis significantly, increasing the complexity of the payoff function formulation.

Furthermore, we could consider modifying our definition of an interview set. Currently we assume that residents could interview up to *k* hospitals for free, but an alternative model to consider would be to allow each resident *r* to have a “budget” $b_r$, and incur a cost, $c_r(h)$, when interviewing hospital *h*, with the constraint that if *S* is the set of hospitals interviewed by resident *r*, then $\sum _{h\in S} c_r(h)\le b_r$. Such a budget, even if the cost is equal for all hospitals, will change the equilibrium in a variety of ways, including by making it no longer always a dominating strategy to interview at all *k* hospitals, as sometime—particularly for very high/low ranked residents—interviewing at some hospitals might not offer enough expected utility. It may also give rise to a setting equivalent to the hospitals having a limited number of potential interview slots, which would make the hospitals strategic players as well, as they wish to find the candidates which are both highly ranked and that will also choose them.^7^ A similar form of either assortative or “reach”/“safety” may happen, this time by the hospitals, though that is outside the scope of this paper.

A long-term research goal is to better understand the extent to which “natural equilibria” exist in matching games, and how such equilibria correspond with observed behaviour in actual markets. One such possibility is for interviewing to be assortative for “safety” programs while allowing for one or a few “reach” programs. (See for example the strategy of resident 6 for small values of the Mallows’ parameter in Fig. 4.) Furthermore, we are interested in techniques that could reduce the cognitive burden placed on participants in matching markets, while also reducing inefficiencies. For example, there may be ways to leverage research on preference elicitation for matching markets (e.g., Drummond and Boutilier 2014) with matching market design so as to guide participants to interview with the appropriate programs so as to improve the overall quality of the match.

## Data Availability

Not applicable.

## Proofs from Sect. 4.1.1

In this appendix we provide the proofs of the results that were presented in Sect. 4.1.1.

### Proof

(Lemma 1) Suppose $\sigma$ is a prefix of $\sigma '$. Then, let $\sigma$ be some ranking with *p* elements, including elements $a_i$ and $a_j$. Let $\sigma '$ be a ranking of $p+1$ elements with $\sigma$ as its prefix, and an additional element $a_{p}$ added at the end. We prove this by starting from the definition of $P(a_i \succ a_j | D ^{\phi ,\sigma '})$, and using algebraic manipulations to show this is equivalent to the definition of $P(a_i \succ a_j | D ^{\phi ,\sigma })$.

2 $$\begin{aligned} P(a_i \succ a_j | D ^{\phi ,\sigma '})&= \frac{\sum _{\eta ' \in \{a_0,\ldots ,a_{p-1},a_p\}_\succ ^{a_i \succ a_j}}\phi ^{d(\eta ',\sigma ')}}{1(1+\phi )\ldots (1+\cdots +\phi ^{p-1} + \phi ^p)} \end{aligned}$$

However, because $a_i,a_j$ are in ranking $\sigma$, the only difference between summing over the set of all rankings in $\{a_0,\ldots ,a_p\}_\succ ^{a_i \succ a_j}$ and $\{a_0,\ldots ,a_{p-1}\}_\succ ^{a_i \succ a_j}$ is that there for each permutation generated by $\{a_0,\ldots ,a_{p-1}\}_\succ$, there are *p* permutations in $\{a_0,\ldots ,a_{p}\}_\succ$, each one with $a_p$ in a different place (and thus a different Kendall-$\tau$ distance). Fixing some $\eta \in \{a_0,\ldots ,a_{p-1}\}_\succ$, if $a_p$ is in the last rank position (as it is in $\sigma '$), the distance is simply $d(\eta ,\sigma )$. If $a_p$ is in the second-to-last position, we have now added in an additional discordant pair, so the distance is $d(\eta ,\sigma )+1$. Using this, we generate the following:

$$\begin{aligned} P(a_i \succ a_j | D ^{\phi ,\sigma '})&= \frac{\sum _{\eta \in \{a_0,\ldots ,a_{p-1}\}_\succ ^{a_i \succ a_j}} \sum _{l = 0}^p \phi ^{d(\eta ,\sigma )+l}}{1(1+\phi )\ldots (1+\ldots + \phi ^p)} \\&= \frac{\big [\sum _{\eta \in \{a_0,\ldots ,a_{p-1}\}_\succ ^{a_i \succ a_j}} \phi ^{d(\eta ,\sigma )}\big ] \big [\sum _{l=0}^p \phi ^l \big ]}{1(1+\phi )\ldots (1+\cdots + \phi ^p)} \\&= \frac{\big [\sum _{\eta \in \{a_0,\ldots ,a_{p-1}\}_\succ ^{a_i \succ a_j}} \phi ^{d(\eta ,\sigma )}\big ](1 + \ldots + \phi ^p)}{1(1+\phi )\ldots (1+\cdots +\phi ^{p-1})(1+\cdots + \phi ^p)} \\&= \frac{\sum _{\eta \in \{a_0,\ldots ,a_{p-1}\}_\succ ^{a_i \succ a_j}} \phi ^{d(\eta ,\sigma )}}{1(1+\phi )\ldots (1+\cdots +\phi ^{p-1})} \\&= P(a_i \succ a_j | D ^{\phi ,\sigma }) \end{aligned}$$

By symmetry, this also holds when $\sigma$ is a suffix of $\sigma '$. $\square$

### Proof

(Corollary 1) Consider $\sigma = a_i \succ a_{i+1}$, a reference ranking with only our two elements in it. Then, the set of all potential rankings such that $a_i \succ a_{i+1}$ under $D ^{\phi ,\sigma }$ is solely the ranking $a_i \succ a_{i+1}$. By the definition of the Mallows model, this ranking has probability $\frac{1}{1+\phi }$. We add some arbitrary prefix $\sigma '$ to $\sigma$ and some arbitrary suffix $\sigma ''$ to $\sigma$ to create a new reference ranking $\gamma$. By Lemma 1, the probability that some $\eta$ is drawn from $D ^{\phi ,\gamma }$ such that $a_i \succ _\eta a_{i+1}$ is $\frac{1}{1+\phi }$ as required. $\square$

### Proof

(Corollary 2) Consider $\sigma ^* = a_i \succ a_{i+1} \succ a_{i+2}$, a reference ranking with three elements in it. The set of all potential rankings under $D ^{\phi ,\sigma ^*}$ such that $a_i \succ a_{i+1} \succ a_{i+2}$ is solely that ranking. Using the same argument as in Lemma 1, we note that creating some new reference ranking $\gamma = \sigma ' \succ \sigma ^* \succ \sigma ''$ and drawing from $D ^{\phi ,\gamma }$ does not change the likelihood that we draw a ranking consistent with $a_i \succ a_{i+1} \succ a_{i+2}$.

Therefore, the probability that we draw a ranking $\beta$ consistent with some permutation $\eta$ of $a_i,a_{i+1},a_{i+2}$ under the distribution $D ^{\phi ,\gamma }$ is simply the probability that we drew $\eta$ under the distribution $D ^{\phi ,\sigma ^*}$, which is $\frac{\phi ^{d(\eta ,\sigma ^*)}}{(1+\phi )(1+\phi +\phi ^2)}$. $\square$

### Proof

(**Lemma** 2) This is equivalent to generating the set of all $(n-1)!$ possible rankings excluding alternative $a_1$ ($a_{n}$), and then adding $a_1$ ($a_{n}$) in place *j*. Whatever the ranking, adding $a_{1}$ ($a_{n}$) in place *j* adds $j-1$ ($n-j$) to each possible ranking’s Kendall’s $\tau$ distance from $\sigma \setminus \{a_{1}\}$ ($\sigma \setminus \{a_{n}\}$), making the distance from $\sigma$ grow by exactly $j-1$ ($n-j$). Similarly, adding $a_{j}$ in first place adds $j-1$ to the distance from $\sigma \setminus \{a_{j}\}$, increasing the distance from $\sigma$ by $j-1$.

However, we also added in an additional element to the ranking (growing from $n-1$ to *n*), and must include that in the normalization factor *Z*. The normalization factor for $n-1$ alternatives is $(1+\phi )(1+\phi ^2)\ldots (1+\cdots +\phi ^{n-2})$. The normalization factor for *n* elements is identical, but multiplied by $1+\cdots +\phi ^{n-1}$. $\square$

### Proof

(Lemma 3) For any $a_{\ell }$, $i>\ell >j$: if $a_{\ell }\succ _{\eta }a_{i}$, this adds at least 1 to the Kendall-$\tau$ distance of $\eta$ from $\sigma$ (due to $a_{i}\succ _{\sigma }a_{\ell }$). But if $a_{i}\succ _{\eta }a_{\ell }$, this means that $a_{j}\succ _{\eta }a_{\ell }$, again adding 1 to the Kendall-$\tau$ distance of $\eta$ from $\sigma$. So the Kendall-$\tau$ distance of $\eta$ from $\sigma$ is at least $\sum _{\ell =i}^{j-1}1=j-i$, and therefore, $P(\eta )<\frac{\phi ^{j-i}}{Z}$. $\square$

## Proofs from Sect. 4.1.2

### Proof

(Lemma 4) The idea behind the proof is that if there is a set of hospitals that are better than interviewing assortatively, since no other resident prior to $r_{k}$ interviews there, the hospitals in this set that are outside of $\{h_{1},\ldots ,h_{k}\}$ have an ordering. That is, the expected utility from adding $h_{k+1}$ is larger than that of adding $h_{k+2}$, since, in expectation $h_{k+1}$ is likely to be ranked higher by the resident than $h_{k+2}$. Therefore, taking out the hospital with the least expected utility from $\{h_{1},\ldots ,h_{k}\}$ and adding $h_{k+1}$ in its stead should already be beneficial, since any other hospital added to the interviewing set will remove a hospital with a higher utility (than the one removed for $h_{k+1}$), and replace it with lesser utility hospital (since an hospital from $h_{k+2},\ldots ,h_{n}$ has smaller expected utility). Therefore, if there is a set that is better than assortative, it should show up already when replacing some hospital in $\{h_{1},\ldots ,h_{k}\}$ by $h_{k+1}$.

For any hospital *h*, let $b(h,\eta ) = 1$ iff *h* is available for $r_{k}$, and $h \succ _\eta h_j$ for all other $h_j$ available; and 0 otherwise. Directly following from the utility function, the utility of resident $r_k$ when interviewing with some set of hospitals $S = \{h_1,\ldots ,h_k\}$ can thus be written as:

$$\begin{aligned} u_{r_k}(S)&= \sum _{h \in S} \sum _{\eta \in H_\succ } v(h,\eta )P(\eta , D ^{\phi ,\sigma })b(h,\eta ) \end{aligned}$$

As we assume knowledge of the strategies for residents $r_1,\ldots ,r_{k-1}$, we can calculate the probability that any given hospital is available. We thus can calculate the contribution of each hospital interview to the total utility, as $P(\eta , D ^{\phi ,\sigma })$ and $v(h,\eta )$ are known a priori. Moreover, when $r_1,\ldots ,r_k$ all interview with the same *k* hospitals, $b(h,\eta )$ is equivalent to the probability that hospital *h* is available for $r_{k}$ (which we denote by *P*(*h*)): resident $r_k$ gets whatever hospital $r_1,\ldots ,r_{k-1}$ do not take.

Now, assume there is in equilibrium some set $S^\prime$ of hospitals such that $u_{r_k}(S^\prime ) > u_{r_k}(S)$. Define $\bar{S} = S {\setminus } S^\prime$; denote the members of $\bar{S}$ as $h^\prime _1,\ldots ,h^\prime _l$. Also, note that $h_{k+1}$ must be in $S^\prime \setminus S$, as $\bar{S} \ne S$ and $h_{k+1}$ dominates all alternatives in $\{h_{k+1},\ldots ,h_n\}$: $h_{k+1}$ is available for $r_k$ with probability 1 (as are all other alternatives not in *S*), and has higher expected value than any other $h_j$ s.t. $h_{k+1} \succ _\sigma h_j$. Without loss of generality, let $h^\prime _1$ be the hospital in $\bar{S}$ that minimizes the benefit gained from swapping some element in $\bar{S}$ with one of the more “desirable” elements in $S^\prime$. More formally, $h^\prime _1$ is the hospital in $\bar{S}$ that minimizes

$$ y_1 = \sum _{\eta \in H_\succ } P(\eta | D ^{\phi ,\sigma })b(h^\prime _1,\eta )\big [v(h^\prime _{1},\eta ) - v(h_{k+1},\eta )\big ] $$

$y_1$ is the value that is lost when $h^\prime _1$ is the only available hospital from $h_1,\ldots ,h_k$, and $h_{k+1}$ must be chosen instead. The value added by interviewing in $h_{k+1}$ instead of $h^\prime _1$ is formally: $z_1 = \sum _{\eta \in H_\succ } P(\eta | D ^{\phi ,\sigma })b(h_{k+1},\eta ) v(h_{k+1},\eta )$. Then, $u_{r_k}(S\cup \{h_{k+1}\}{\setminus } \{h^\prime _1\}) = u_{r_k}(S) - y_1 + z_1$. If $y_1 \le z_1$, the lemma is proven; Otherwise, we assume $z_1-y_{1}<0$ and establish a contradiction.

Without loss of generality, let $h^\prime _2$ be the hospital in $\bar{S} \setminus \{h^\prime _1\}$ that minimizes

$$\begin{aligned} y_2 =&\sum _{\eta \in H_\succ } P(\eta | D ^{\phi ,\sigma })b(h^\prime _2,\eta )\big [v(h^\prime _{2},\eta ) - \max (v(h_{k+1},\eta ),v(h_{k+2},\eta ))\big ]\\ =&\sum _{\eta \in H_{\succ | h_{k+1}\succ h_{k+2}}} P(\eta | D ^{\phi ,\sigma })b(h^\prime _2,\eta )\big [v(h^\prime _{2},\eta ) -v(h_{k+1},\eta )\big ] \\&\quad +\sum _{\eta \in H_{\succ | h_{k+2}\succ h_{k+1}}} P(\eta | D ^{\phi ,\sigma })b(h^\prime _2,\eta )\big [v(h^\prime _{2},\eta ) -v(h_{k+2},\eta )\big ] \end{aligned}$$

Again, $y_2$ is the benefit we get from $h^\prime _2$, the alternative we are swapping out for $h_{k+2}$. The value added from $h_{k+2}$ is $z_2 = \sum _{\eta \in H_\succ } P(\eta | D ^{\phi ,\sigma })b(h_{k+2},\eta )v(h_{k+2})$. Since $h_{k+1}$ and $h_{k+2}$ have the same probability of being available, but the expected value of $v(h_{k+1})$ is more than that of $v(h_{k+2})$, we know $z_{2}<z_{1}$. Thanks to Corollary 1:

$$ \sum _{\eta \in H_{\succ | h_{k+1}\succ h_{k+2}}} P(\eta | D ^{\phi ,\sigma })b(h^\prime _2,\eta )\big [v(h^\prime _{2},\eta ) -v(h_{k+1},\eta )\big ]=\frac{1}{1+\phi }y_{2} $$

Looking at the equivalent section of $y_{1}$:

$$ \sum _{\eta \in H_{\succ | h_{k+1}\succ h_{k+2}}} P(\eta | D ^{\phi ,\sigma })b(h^\prime _1,\eta )\big [v(h^\prime _{1},\eta ) -v(h_{k+1},\eta )\big ]>\frac{1}{1+\phi }y_{1} $$

but thanks to $y_{1}$ minimality:

$$\begin{aligned}&\sum _{\eta \in H_{\succ | h_{k+1}\succ h_{k+2}}} P(\eta | D ^{\phi ,\sigma })b(h^\prime _2,\eta )\big [v(h^\prime _{2},\eta ) -v(h_{k+1},\eta )\big ] \\&\quad >\sum _{\eta \in H_{\succ | h_{k+1}\succ h_{k+2}}} P(\eta | D ^{\phi ,\sigma })b(h^\prime _1,\eta )\big [v(h^\prime _{1},\eta ) -v(h_{k+1},\eta )\big ] \end{aligned}$$

and therefore $y_{2}>y_{1}$. Thus:

$$\begin{aligned} u_{r_k}(S \setminus \{h^\prime _1,h^\prime _2\} \cup \{h_{k+1},h_{k+2}\})&= u_{r_k}(S) - y_1 + z_1 - y_2 + z_2 \\&< u_{r_k}(S) - 2y_1 + 2z_1 \\&< u_{r_k}(S) \end{aligned}$$

Note that due to similar considerations, all other alternatives in $S \setminus S^\prime$ must also have $y_i$ such that $y_i > y_1$ and $z_i < z_1$, by the construction of $y_1$ and $z_1$. Let $l = |\bar{S}|$. Thus:

$$\begin{aligned} u_{r_k}(S^\prime )&= u_{r_k}(S \setminus \bar{S}) + \sum _{i=1}^l z_i - y_i< u_{r_k}(S) - ly_1 + lz_1 < u_{r_k}(S) \end{aligned}$$

This contradicts our assumption that $u_{r_k}(S^\prime ) > u_{r_k}(S)$; thus, if such an $S^{\prime }$ exists, $y_1 \ge z_1$, and showing that *S* dominates $S {\setminus } \{h_j\} \cup \{h_{k+1}\}$ is sufficient for all $h_j \in S$. $\square$

### Proof

(Lemma 5) By Lemma 4, showing that the marginal contribution from $h_j$ is bigger than the marginal contribution from $h_{k+1}$ is sufficient to show that *S* dominates any other interviewing set. Using the payoff function in Sect. 3.1, this means that we want to find conditions such that the utility to $r_k$ provided by $h_j$ is larger than that of $h_{k+1}$:

3 $$\begin{aligned} \sum _{\eta \in H_\succ } v(h_j,\eta ) P(\mu (h_j)&= r_k | S,\eta , D ^{\phi ,\sigma }) P(\eta | D ^{\phi ,\sigma }) \ge \nonumber \\&\sum _{\eta \in H_\succ } v(h_{k+1},\eta ) P(\mu (h_{k+1}) = r_k | S^\prime ,\eta , D ^{\phi ,\sigma }) P(\eta | D ^{\phi ,\sigma }) \end{aligned}$$

Note that, when interviewing with set *S*, the probability $\mu (h_j) = r_k$ is simply the probability that no resident in $r_1,\ldots ,r_{k-1}$ chooses $h_j$. Thus, the left hand side of Eq. 3 simplifies to:

4 $$\begin{aligned} \sum _{\eta \in H_\succ } v(h_j,\eta ) P(\mu (h_j)&= r_k | S,\eta , D ^{\phi ,\sigma } )P(\eta | D ^{\phi ,\sigma })\nonumber \\&= P(h_j)\sum _{\eta \in H_\succ } v(h_j,\eta ) P(\eta | D ^{\phi ,\sigma }) \nonumber \\&= P(h_j)\mathbb {E}(v(h_j) | D ^{\phi ,\sigma }) \end{aligned}$$

We now also wish to simplify the right hand side. Note that there are two cases in which resident $r_k$ is matched with $h_{k+1}$ when interviewing with set $S^\prime$: either $h_j$ is the only hospital available (i.e., $r_1,\ldots ,r_{k-1}$ have all been matched with $\{h_1,\ldots ,h_k\} \setminus \{h_j\}$), or for some $h_i \in \{h_1,\ldots ,h_k\}{\setminus } \{h_j\}$, $h_i$ is available and under the ranking $\eta$ in consideration, $h_{k+1} \succ _\eta h_i$. Again, $\mathbb {1}(y)$ denote an indicator function, where $\mathbb {1}(y) = 1$ iff *y* is true, and 0 otherwise. More formally, we express the RHS of the condition in Eq. 3 using the indicator function, and simplify:

5 $$\begin{aligned} \sum _{\eta \in H_\succ }&P(\eta | D ^{\phi ,\sigma }) \cdot \big [ v(h_{k+1},\eta )P(h_j) +\nonumber \\&\quad \quad \sum _{h_i \in S^\prime } P(h_i)\mathbb {1}(h_{k+1} \succ _\eta h_i)v(h_{k+1},\eta )\big ]= \nonumber \\&=P(h_j) \mathbb {E}(v(h_{k+1}) | D ^{\phi ,\sigma }) \nonumber \\&\quad + \sum _{\eta \in H_\succ } P(\eta | D ^{\phi ,\sigma })\cdot \big [\sum _{h_i \in S^\prime } P(h_i)\mathbb {1}(h_{k+1} \succ _\eta h_i)v(h_{k+1},\eta ) \big ] \end{aligned}$$

Combining the simplifications provided in Eqs. 4 and 5 completes the proof. $\square$

## Sufficient inequality for checking assortativity

We provide a simplified condition for assortative interviewing that is sufficient though not necessary. This condition is easier to compute than the condition in Lemma 5, and thus will be valuable later on, when verifying whether specific valuation functions admit assortative interviewing equilibria.

### Lemma 8

Given an interviewing quota of *k* interviews, a dispersion parameter $\phi$, and a scoring function *v*, if residents $r_1,\ldots ,r_{k-1}$ all interview assortatively (i.e., with hospital set $S = \{h_1,\ldots ,h_{k}\}$), then satisfying the following inequality for all $h_j \in \{h_1,\ldots ,h_k\}$ when $S^\prime = S \setminus \{h_j\} \cup \{h_{k+1}\}$ is sufficient to show that assortative interviewing is a best response for resident $r_k$:

$$\begin{aligned} P(h_j )\mathbb {E}(v(h_j) | D ^{\phi ,\sigma })&\ge P(h_j ) \mathbb {E}(v(h_{k+1}) | D ^{\phi ,\sigma }) \nonumber \\&+ \sum _{h_i \in S^\prime } P(h_i ) \mathbb {E}(v(h_k^{\prime }) | D ^{\phi ,\sigma ^\prime }) \frac{\phi }{Z(1-\phi )} \end{aligned}$$

where $\sigma ^\prime$ is equivalent to the reference ranking $\sigma$ with one element $h_i$ s.t. $h_j \succ _\sigma h_i$ removed, and $h'_{k}$ is the *k*th item in $\sigma '$.

### Proof

We begin from the sufficient and necessary condition stated in Lemma 5. Note that we can generate any ranking such that $h_{k+1} \succ h_i$ (for some given *i*) by iterating over all permutations of $H \setminus \{h_i\}$, and for each permutation, placing $h_{i}$ in every slot below $h_{k+1}$. There are at most $n-1$ slots that $h_i$ could be placed in.

Let $\sigma ^\prime$ be identical to the reference ranking $\sigma$, except with $h_i$ removed. Rename every element after $h_i$ such that it corresponds to its current index: in other words, $h^\prime _{j} = h_{j+1}$ for all $j \ge i$. Let $\eta ^\prime$ be some arbitrary ranking drawn from $D ^{\phi ,\sigma ^\prime }$. Let $H^\prime = H \setminus \{h_i\}$. Remember, $S^\prime = \{h_1,\ldots ,h_{k+1}\} {\setminus } \{h_j\}$. Thus, we note that:

$$\begin{aligned} \sum _{\eta \in H_\succ }&\sum _{h_i \in S^\prime } P(h_i )\mathbb {1}(h_{k+1} \succ _\eta h_i)v(h_{k+1},\eta )P(\eta | D ^{\phi ,\sigma }) \nonumber \\&\le \sum _{h_i \in S^\prime } \left[ P(h_i ) \left( \sum _{\eta ^\prime \in H^\prime _\succ } v(h_k^{\prime },\eta ^\prime ) P\left( \eta ^{\prime } | D ^{\phi ,\sigma ^{\prime }}\right) \left( \sum _{l=1}^{n} \frac{\phi ^l}{Z} \right) \right) \right] \end{aligned}$$

However, note that $\phi ^l$ is a geometric series. We let $n \rightarrow \infty$, giving us:

6 $$\begin{aligned} \sum _{h_i \in S^\prime }&\left[ P(h_i ) \mathbb {E}\left( v(h_k^{\prime }\right) | D ^{\phi ,\sigma ^\prime }) \sum _{l=1}^{n} \frac{\phi ^l}{Z}\right] \le \sum _{h_i \in S^\prime } P(h_i ) \mathbb {E}(v(h_k^{\prime }) | D ^{\phi ,\sigma ^\prime }) \frac{\phi }{Z(1-\phi )} \end{aligned}$$

Thus, because Eq. 6 is an upper bound, it is sufficient to show the following, as required:

$$\begin{aligned} P(h_j )\mathbb {E} \left( v(h_j) | D ^{\phi ,\sigma }\right)&\ge P(h_j ) \mathbb {E} \left( v(h_{k+1}) | D ^{\phi ,\sigma }\right) \nonumber \\&\quad + \sum _{h_i \in S^\prime } P(h_i ) \mathbb {E} \left( v\left( h_k^{\prime }\right) | D ^{\phi ,\sigma ^\prime }\right) \frac{\phi }{Z(1-\phi )} \end{aligned}$$

$$\square $$

## Proofs from Sect. 5

Before proving the results in this section we introduce some notation. Given some resident with ranking $\eta$ over hospitals, define $s_i$ to be the *i*’th ranked hospital in this list. While in general we hesitate to introduce new notation, it is important to distinguish between some hospital in *H* and the *i*’th ranked one from a resident’s perspective.

### Proof

(Lemma 6) We begin with the condition in Lemma 5:

$$\begin{aligned} P(h_j)\mathbb {E} \left( v(h_j) | D ^{\phi ,\sigma }\right)&> P(h_j) \mathbb {E} \left( v(h_{k+1}) | D ^{\phi ,\sigma }\right) + \\&\sum _{\eta \in H_\succ } P(\eta | D ^{\phi ,\sigma }) \cdot \left[ \sum _{h_i \in S^\prime } P(h_i)\mathbb {1}(h_{k+1} \succ _\eta h_i)v(h_{k+1},\eta ) \right] \\ \end{aligned}$$

We instantiate this condition for the plurality function, noting that $v(h,\eta ) > 0$ iff *h* is top-ranked in $\eta$.

$$\begin{aligned}&P(h_j )\mathbb {E} \left( v(h_{k+1})| D ^{\phi ,\sigma }\right) + \sum _{i=1}^{j-1} P(h_i ) \mathbb {E}\left( v(h_{k+1}) | D ^{\phi ,\sigma }\right) \\&\quad + \sum _{i=j+1}^{k} P(h_i ) \mathbb {E}\left( v(h_{k+1}) | D ^{\phi ,\sigma }\right) = \sum _{i = 1}^k P(h_i) \mathbb {E}\left( v(h_{k+1}) | D ^{\phi ,\sigma }\right) \end{aligned}$$

But, again, as the expected value for any hospital *h* is simply the probability that *h* is $s_1$ this further simplifies to:

$$\begin{aligned} \nonumber P(h_{k+1}=s_1)\sum _{i=1}^k P(h_i ) = P(h_{k+1}= s_1) \end{aligned}$$

Note that $\sum _{i=1}^k P(h_i) = 1$ as all residents $r_1,\ldots , r_{k-1}$ have been matched with exactly $k-1$ hospitals in $h_1,\ldots ,h_k$, leaving exactly one hospital left with probability 1.

Applying Lemma 2 to both sides of the inequality (recall that $\mathbb {E}(v(h_{j})| D ^{\phi ,\sigma })$ is simply $P(h_{j}=s_1)$):

$$\begin{aligned} P(h_j)\frac{\phi ^{j-1}}{1+\cdots +\phi ^{n-1}} \ge&\frac{\phi ^k}{1+\cdots +\phi ^{n-1}}\\ \\ P(h_j ) \ge&\phi ^{k-j+1} \end{aligned}$$

$$\square $$

### Proof

(Lemma 7) Looking at the condition of Lemma 5

$$\begin{aligned} P(h_j )\mathbb {E}(v(h_j) | D ^{\phi ,\sigma })&\ge P(h_j ) \mathbb {E}\left( v(h_{k+1}) | D ^{\phi ,\sigma }\right) + \\&\sum _{\eta \in H_\succ } P\left( \eta | D ^{\phi ,\sigma }\right) \left[ \sum _{h_i \in S^\prime } P(h_i)\mathbb {1}(h_{k+1} \succ _\eta h_i)v(h_{k+1},\eta ) \right] \end{aligned}$$

We will first expand the value expectation ($\mathbb {E}$):

$$\begin{aligned} P(h_j )\sum _{i=1}^n P(h_j =s_i)v(s_i)&\ge P(h_j) \sum _{i=1}^n P(h_{k+1} = s_i)v(s_i) \\&+ \sum _{\begin{array}{c} \eta \in H_\succ |\\ h_{k+1} = s_{1} \end{array}} P(\eta | D ^{\phi ,\sigma }) \sum _{h_i \in S^\prime } P(h_i)\mathbb {1}(h_{k+1} \succ _\eta h_i)v(s_{1}) \\&+\cdots +\sum _{\begin{array}{c} \eta \in H_\succ |\\ h_{k+1}= s_{n-1} \end{array}} P(\eta | D ^{\phi ,\sigma }) \sum _{h_i \in S^\prime } P(h_i)\mathbb {1}(h_{k+1} \succ _\eta h_i)v(s_{n-1}) \end{aligned}$$

Note that for any $1\le \ell \le n$,

$$\begin{aligned} v(s_{\ell })>P&(h_j)P(h_{j}=s_{\ell })v(s_{\ell })+\\&\sum _{\begin{array}{c} \eta \in H_\succ |\\ h_{k+1} = s_{\ell } \end{array}} P(\eta | D ^{\phi ,\sigma }) \sum _{h_i \in S^\prime } P(h_i )\mathbb {1}(h_{k+1} \succ _\eta h_i)v(s_{\ell }) \end{aligned}$$

Thus, combining these and Lemma 5, it is sufficient to show the following holds whenever plurality admits an assortative interviewing equilibrium:

7 $$\begin{aligned} P(h_j)P(h_{j}=s_{1})v(s_{1})\ge P(h_j)P(h_{k+1}=s_{1})v(s_{1})+\sum _{\ell =2}^{n}v(s_{\ell }) \end{aligned}$$

We assume that for plurality valuation, the condition has a strict inequality. In other words:

$$ P(h_j)P(h_{j}=s_{1})> P(h_j)P(h_{k+1}=s_{1}) $$

Hence, there is an $\bar{\epsilon }\le 1$ such that for all $1\le j\le k$,

$$ P(h_j )P(h_{j}{\text { in }}\,s_{1})-\bar{\epsilon }> P(h_j )P(h_{k+1}{\text { in }}\,s_{1}) $$

Now, for $\epsilon <\frac{\bar{\epsilon }}{2}$, examine the valuation function $v(s_{\ell })=\epsilon ^{\ell -1}$. Note that $\sum _{\ell =2}^{n}\epsilon ^{\ell -1}\le \sum _{\ell =1}^{\infty }\epsilon ^{\ell }=\frac{\epsilon }{1-\epsilon }\le 2\epsilon$. This simplifies such that it satisfies Eq. 7, as required:

$$\begin{aligned} P(h_j )P(h_{j}=s_{1})&> P(h_j )P(h_{k+1}=s_{1})+2\epsilon \\&\ge P(h_j )P(h_{k+1}=s_{1})+\sum _{\ell =2}^{n}v(s_{\ell }) \end{aligned}$$

$$\square $$

### Proof

(Theorem 4) We begin by using the condition from Lemma 6 for $h_{1}$. We thus wish to show conditions on $\phi$ s.t. $P(h_1 )~\ge ~\phi ^2$, when resident $r_2$ chooses their interview set. For $r_2$, $h_1$ is available iff $r_1$ happened to draw a ranking over their preferences s.t. $h_2 \succ h_1$. Then, by Corollary 1, $P(h_1 ) = \frac{\phi }{1 + \phi }$, implying we need to satisfy the equation $\frac{\phi }{1 + \phi } \ge \phi ^2$, which is true whenever $0 < \phi \le 0.6180$. Doing the same for $h_2$ provides a bound of $0 < \phi \le 0.7549$, so we take the tighter bound of 0.618. $\square$

### Proof

(Theorem 5) We begin by noting that, because of Lemma 8, we only need to show that assortative interviewing is an equilibrium when $0 < \phi \le 0.265074$ for resident $r_2$, and it will hold for all $r_i$. Furthermore, by Lemma 4, we only need to prove that $\{h_1,h_2\}$ dominates both $\{h_1,h_3\}$ and $\{h_2,h_3\}$ to show that it dominates all other possible interviewing sets of size 2.

We prove that choosing $\{h_1,h_2\}$ is better than choosing $\{h_2,h_3\}$, for all values of $\phi$ such that $0 < \phi \le 0.265074$. We prove this by summing over all possible preference rankings that induce a specific permutation of the alternatives $h_1,h_2,h_3$. We then pair these summed permutations in such a manner that makes it easy to find a lower bound for $u_{r_2}(\{h_1,h_2\}) - u_{r_2}(\{h_2,h_3\})$. This lower bound is entirely in terms of $\phi$, meaning that for any $\phi$ such that this bound is above 0, it will be above 0 for any market size *n*.

We look at three cases, pairing all possible permutations of $h_1,h_2,h_3$ as follows:

**Case 1:** all rankings $\eta$ consistent with $h_2 \succ h_1 \succ h_3$ or $\eta '$ consistent with $h_2 \succ h_3 \succ h_1$;

**Case 2:** all rankings $\eta$ consistent with $h_1 \succ h_2 \succ h_3$ or $\eta '$ consistent with $h_3 \succ h_2 \succ h_1$;

**Case 3:** all rankings $\eta$ consistent with $h_1 \succ h_3 \succ h_2$ or $\eta '$ consistent with $h_3 \succ h_1 \succ h_2$.

Note that as we have enumerated all possible permutations of $h_1, h_2, h_3$, these three cases generate every ranking in $H_\succ$. Furthermore, for any one of the three cases, we can iterate only over all possible rankings $\eta$ that are consistent with the first member of the pair, and generate the ranking $\eta '$ consistent with the second member of the pair by simply swapping two alternatives in the rank. Moreover, given some $\eta$, the number of discordant pairs in $\eta '$ is simply the number of discordant pairs in $\eta$, plus the number of additional discordant pairs between $h_1,h_2,h_3$ caused by swapping the two alternatives.

For clarity, let $u_{r_2}(\{h_1,h_2\}) - u_{r_2}(\{h_2,h_3\}) = U_1 + U_2 + U_3$, where $U_1,U_2,U_3$ correspond to our three cases. We also introduce the notation $P_{\mu (r_i)}(h)$ to denote the probability that $r_i$ is matched to hospital *h* under matching $\mu$. That is, $P_{\mu (r_i)}(h)=P(\mu (r_i)=h)$.

**Case 1.** Because we have fixed $h_2 \succ h_1 \succ h_3$ or $h_2 \succ h_3 \succ h_1$, we know exactly what $r_2$’s match will be. As we know $r_1$’s interviewing set ($\{h_1,h_2\}$), and the distribution $r_1$’s preferences are drawn *i.i.d.*, we know the likelihood that either $h_1$ or $h_2$ is available; by Lemma 1, $P(\mu (r_1) = h_1) = \frac{1}{1+\phi }$. Using this information, the payoff function, and the definition of $\eta ,\eta '$, we find a lower bound:

8

**Case 2.** We fix $h_1 \succ h_2 \succ h_3$ or $h_3 \succ h_2 \succ h_1$. This case is analogous to Case 1:

9

**Case 3.** We fix $h_1 \succ h_3 \succ h_2$ or $h_3 \succ h_1 \succ h_2$. Again, we look at pairs of rankings $\eta ,\eta '$, where $\eta$ is consistent with $h_1 \succ h_3 \succ h_2$, and $\eta '$ is identical to $\eta$, except ${\text {rank}}\,(h_1,\eta ) = {\text {rank}}\,(h_3,\eta ')$, and ${\text {rank}}\,(h_3,\eta ) = {\text {rank}}\,(h_1,\eta ')$.

Then, as before, we sum over all possible rankings consistent with $h_1 \succ h_3 \succ h_2$, but we break this into two subcases, so that $U_3 = U_{3a} + U_{3b}$:

Case $U_{3b}$ is similar to Cases 1 and 2:

10

Case $U_{3a}$, however, is different from all other cases, in that *all* terms are negative. We note that $U_{3a}$ as above is a monotonically decreasing function in terms of *n*. Thus, if $U_{3a}$ converges as $n \rightarrow \infty$, we have found a lower bound for all *n*. Using this technique, we show the following bound holds:

11 $$\begin{aligned} U_{3a} \ge P_{\mu (r_1)}(h_1) \frac{-\phi }{(1+\phi )(1+\phi +\phi ^2)}\big (\frac{\phi }{(1-\phi )^4}+\frac{1}{3(1-\phi )^3}+\frac{2}{3}\big )(1+\phi ) \end{aligned}$$

We have considered all cases, and can now combine them together. We add the bounds for $U_1$ (Eq. 8), $U_2$ (Eq. 9), $U_{3a}$ (Eq. 11), and $U_{3b}$ (Eq. 10). We simplify using Corollaries 1 and 2, giving us:

12 $$\begin{aligned}&{u_{r_2}(\{h_1,h_2\}) - u_{r_2}(\{h_2,h_3\}) } \nonumber \\&\quad {\ge \frac{\phi ^2}{(1+\phi )(1+\phi )(1+\phi +\phi ^2)}(1-\phi ) +\frac{2(\phi - \phi ^3 - \phi ^4)}{(1+\phi )(1+\phi )(1+\phi +\phi ^2)} } \nonumber \\&\qquad { -\frac{\phi }{(1+\phi )(1+\phi )(1+\phi +\phi ^2)}\big (\frac{\phi }{(1-\phi )^4} + \frac{1}{3(1-\phi )^3}+\frac{2}{3}\big )(1+\phi ) } \nonumber \\&\qquad { +\frac{\phi ^2}{(1+\phi )(1+\phi )(1+\phi +\phi ^2)}(1-\phi )} \end{aligned}$$

Thus, Eq. 12 gives us a lower bound for the difference in expected utility between $\{h_1,h_2\}$ and $\{h_2,h_3\}$ for resident $r_2$, for all *n*. Using numerical methods to approximate the roots of Eq. 12, we get that there is a root at 0, and a root at $\phi \approx 0.265074$.

As the calculations are analogous, we omit the discussion of their derivation, but it can be shown that:

13 $$\begin{aligned}&{u_{r_2}(\{h_1,h_2\}) - u_{r_2}(\{h_1,h_3\}) } \ge \frac{1}{(1+\phi )(1+\phi +\phi ^2)}\nonumber \\&\quad \times {\big [1+\phi -2\phi ^2 - 2\phi ^3 -2\phi ^3\big (\frac{\phi }{(1-\phi )^4} + \frac{1}{3(1-\phi )^3} + \frac{2}{3}\big )\big ]} \end{aligned}$$

Using numerical methods, it can be shown that this is positive for $0< \phi < 0.413633$.

Thus, for the interval $0 < \phi \le 0.265074$, we have shown that $r_2$’s best move in this interval is to interview with $\{h_1,h_2\}$. Then, by Lemma 8, this is an equilibrium for all $r_i$ as required. $\square$

### Proof

(Theorem 6) We provide a counterexample for $n = 4$, $k = 3$. Suppose residents $r_1$ and $r_2$ interview assortatively, both interviewing with $S = \{h_1,h_2,h_3\}$. We show that for resident $r_3$, interviewing with interviewing set $S^\prime = \{h_2,h_3,h_4\}$ dominates interviewing with $S = \{h_1,h_2,h_3\}$ for all $\phi$.

By Lemma 4, it is sufficient to show that if the marginal value in interviewing with $h_4$ dominates the marginal value in interviewing with $h_1$ (as these two sets only differ by these two items), then interviewing with $\{h_2,h_3,h_4\}$ dominates $\{h_1,h_2,h_3\}$. We thus instantiate Eq. 3 for $n = 4$, $k = 3$, *S*, and $S^\prime$ as above for resident $r_3$. Note that $Z = (1+\phi )(1+\phi +\phi ^2)(1+\phi +\phi ^3)$. Let $\mathbb {E}(u(h_i,S))$ denote the expected marginal value in interviewing alternative $h_i$ in set *S*; remember $v(s_i) = 5-i$.

14 $$\begin{aligned} \mathbb {E}(u(h_1,S))&= \sum _{\eta \in H_\succ } v(h_1,\eta ) P(\mu (h_1) = r_3 | S,\eta , D ^{\phi ,\sigma }) P(\eta | D ^{\phi ,\sigma }) \\ \mathbb {E}(u(h_4,S^\prime ))&= \sum _{\eta \in H_\succ } v(h_4,\eta ) P(\mu (h_4) = r_3 | S^\prime ,\eta , D ^{\phi ,\sigma }) P(\eta | D ^{\phi ,\sigma }) \end{aligned}$$

As before, Eq. 14 is simply the probability that $h_1$ is available times the expected value of $h_1$. As noted, $\mathbb {E}(v(h_1) | D^{\phi ,\sigma }) = \sum _{i=1}^4 P(h_1 {\text { in }}\, s_i)\cdot v(s_i) = \sum _{i=1}^4 P(h_1 {\text { in }}\, s_i) \cdot (5-i)$. However, using Lemma 2, we know that $P(h_1 {\text { in }}\, s_i) = \frac{\phi ^{i - 1}}{1+\phi +\phi ^2+\phi ^3}$, giving:

15 $$\begin{aligned} \mathbb {E}(u(h_1,S)) = P(h_1 )\mathbb {E}(v(h_1) | D ^{\phi ,\sigma }) = P(h_1 )\frac{4+3\phi +2\phi ^2+\phi ^3}{1+\phi +\phi ^2+\phi ^3} \end{aligned}$$

Let $P(h_i {\text { taken}})$ denote the probability that either $r_1$ is matched to $h_i$, or $r_2$ is matched to $h_i$ (i.e., $h_i$ is taken by the time we get to resident $r_3$). Also let $P(\mu (r_3) = h_4 | h_4 {\text { in }}\, s_i)$ denote the probability that $r_3$ is matched to $h_4$ if $h_4$ is in slot $s_i$ in $r_3$’s ranking. This is easily calculable by enumerating over the subset of possible rankings such that this occurs, given that $r_1$ and $r_2$ have already taken certain alternatives. Then, using Lemma 2 again and an analogous approach as above, we adapt Eq. 15:

16 $$\begin{aligned} \mathbb {E}(u(h_4,S^\prime ))&= \sum _{i=1}^4 v(s_i)P(h_4 {\text { in }}\, s_i)P(\mu (r_3) = h_4 | h_4 {\text { in }}\, s_i) \nonumber \\&= \frac{4\phi ^3}{1+\phi +\phi ^2+\phi ^3} \nonumber \\&\quad + \frac{3}{Z}\big (\phi ^2+\phi ^3+P(h_2 {\text { taken}}\,)(\phi ^3+\phi ^4) + P(h_3 {\text { taken}}\,)(\phi ^4+\phi ^5)\big ) \nonumber \\&\quad + \frac{2}{Z}\big (P(h_2 {\text { taken}}\,)(\phi +\phi ^2) + P(h_3 {\text { taken}}\,)(\phi ^2 + \phi ^3) + P(h_1 )(\phi ^3 +\phi ^4)\big ) \nonumber \\&\quad + \frac{P(h_1 )}{1+\phi +\phi ^2+\phi ^3} \end{aligned}$$

As we assume that residents $r_1$ and $r_2$ both interview with *S*, the probability that $h_1$ is available, or $h_2$ (resp. $h_3$) is taken is the same across both $\mathbb {E}(u(h_1,S))$ and $\mathbb {E}(u(h_4,S^\prime ))$. We instantiate these as follows, by determining the probability that $r_1$ is matched to some hospital $h_j$ other than $h^*$, and enumerate the probabilities of all rankings such that $r_2$ is matched to some hospital $h_j^\prime \ne h^*$ given that $r_1$ is matched to $h_j$:

$$\begin{aligned} {\scriptstyle P(h_1 ) }&{\scriptstyle = P(\mu (r_1) = h_2 | S, D ^{\phi ,\sigma })(\frac{\phi ^2}{1+\phi +\phi ^2+\phi ^3} + \frac{\phi ^2+\phi ^3+\phi ^4+2\phi ^5+\phi ^6}{Z}) } \nonumber \\&{\scriptstyle \quad + P(\mu (r_1) = h_3 | S, D ^{\phi ,\sigma })(\frac{\phi }{1+\phi +\phi ^2+\phi ^3} + \frac{\phi ^3+2\phi ^4+2\phi ^5+\phi ^6}{Z})} \\ {\scriptstyle P(h_2 {\text { taken}}\,) }&{\scriptstyle = P(\mu (r_1) = h_2 | S, D ^{\phi ,\sigma }) + P(\mu (r_1) = h_3 | S, D ^{\phi ,\sigma })(\frac{\phi }{1+\phi +\phi ^2+\phi ^3} + \frac{\phi ^3+2\phi ^4+2\phi ^5+\phi ^6}{Z}) }\nonumber \\&{\scriptstyle \quad + P(\mu (r_1) = h_1 | S, D ^{\phi ,\sigma })(\frac{\phi }{1+\phi +\phi ^2+\phi ^3} + \frac{1+\phi +\phi ^2+\phi ^3+\phi ^4+\phi ^5}{Z})}\\ {\scriptstyle P(h_3 {\text { taken}}\,) }&{\scriptstyle = P(\mu (r_1) = h_3 | S, D ^{\phi ,\sigma }) + P(\mu (r_1) = h_2)(\frac{\phi ^2}{1+\phi +\phi ^2+\phi ^3} + \frac{\phi ^2+\phi ^3+\phi ^4+2\phi ^5+\phi ^6}{Z})} \nonumber \\&{\scriptstyle \quad + P(\mu (r_1) = h_1 | S, D ^{\phi ,\sigma })(\frac{\phi ^2}{1+\phi +\phi ^2+\phi ^3} + \frac{1+\phi +\phi ^2+\phi ^3+\phi ^4+\phi ^5+\phi ^6}{Z}) } \end{aligned}$$

It is also possible to calculate exact values for the probability that $r_1$ is matched to $h_1,h_2,h_3$. We do this by calculating the probability that alternative is first, or the probability that alternative is second, and $h_4$ is first:

$$\begin{aligned} P(\mu (r_1) = h_1 | S, D ^{\phi ,\sigma })&= P(h_1 {\text { in }}\, s_1) + P(h_1 {\text { in }}\, s_2 {\text { and }}\, h_4 {\text { in }}\, s_1) \\&= \frac{1}{1+\phi +\phi ^2+\phi ^3} + \frac{\phi ^3 + \phi ^4}{Z} \\ P(\mu (r_1) = h_2 | S, D ^{\phi ,\sigma })&=P(h_2 {\text { in }}\, s_1) + P(h_2 {\text { in }}\, s_2 {\text { and }}\, h_4 {\text { in }}\, s_1) \\&= \frac{\phi }{1+\phi +\phi ^2+\phi ^3} + \frac{\phi ^4 + \phi ^5}{Z} \\ P(\mu (r_1) = h_3 | S, D ^{\phi ,\sigma })&=P(h_3 {\text { in }}\, s_1) + P(h_3 {\text { in }}\, s_2 {\text { and }}\, h_4 {\text { in }}\, s_1)\\&= \frac{\phi ^2}{1+\phi +\phi ^2+\phi ^3} + \frac{\phi ^5 + \phi ^6}{Z} \end{aligned}$$

By combining the equations for the probabilities we are left with two equations depending only on $\phi$. Moreover, after instantiating $\mathbb {E}(u(h_1,S))$ and $\mathbb {E}(u(h_4,S^\prime )$ above, we note that both functions are continuous on the interval (0, 1]. Using numerical techniques, it can be shown that there are no zeros for the function $\mathbb {E}(u(h_1,S)) - \mathbb {E}(u(h_4,S^\prime ))$ on the interval (0, 1], and the function is negative on the interval (0, 1] providing the counterexample as required. $\square$

### Proof

(Theorem 7) For $k = 3$, we simply check the constraint

$$ P(h_j)\ge \phi ^{k-j+1} $$

from Lemma 6 with $h_j = h_1,h_2,h_3$. We find that the marginal contribution from $h_1$ is less than the marginal contribution of $h_2$ or $h_3$, and thus only present the calculation for $h_1$. We directly compute $P(h_1)$, by multiplying the probability that $r_1$ did not take $h_1$, and multiplying it by the probability that $r_2$ did not take $h_1$, given that $r_1$ *also* did not take $h_1$. To calculate this we enumerate the probabilities of any possible rankings:

17 $$\begin{aligned} P(h_1 )&= P(\mu (r_1) \ne h_1)P(\mu (r_2) \ne h_1 | \mu (r_1) \ne h_1) \nonumber \\ P(h_1)&= (\frac{\phi + 2\phi ^2 + \phi ^3}{(1+\phi )(1+\phi +\phi ^2)})(\frac{\phi ^2+2\phi ^3}{(1+\phi +\phi ^2)}) \nonumber \end{aligned}$$

The first parenthesis is using Corollary 2, and the second the probability $h_{3}$ is preferred over $h_{2}$ using Corollary 1. Using numerical methods to find the roots of $P(h_1 ) - \phi ^3$, we can show that above constraint holds when $0 < \phi \le 0.5462$. $\square$

### Assortative equilibria when $k \ge 4$

We start by providing an additional lemma regarding a bound on the availability of any given alternative $h_i$ at the time resident $r_k$ is being matched by the mechanism to their favourite remaining hospital. This probability is dependent on $\phi$: for any hospital $h_i$ such that $i < k$, as $\phi \rightarrow 1$, the probability $h_i$ is available goes to $\frac{1}{k}$; as $\phi \rightarrow 0$, this probability goes to 0. Instead of looking at the probability directly, we look at the probability that a preference profile will admit a stable match such that $h_i$ is available, and bound that.

#### Lemma 9

Given a Mallows model with dispersion parameter $\phi$, assortative interviewing for residents $r_1,\ldots .,r_{k-1}$, and a hospital $h_i \in \{h_1,\ldots ,h_k\}$ (i.e., the residents’ interview set), then any profile $\eta _{1},\ldots ,\eta _{k-1}\in D ^{\phi ,\sigma }$ of $k-1$ preferences (for $r_{1},\ldots ,r_{k-1}$) such that $h_{i}$ is available for $r_{k}$ has probability $P(r_{1}=\eta _{1},r_{2}=\eta _{2},\ldots ,r_{k-1}=\eta _{k-1}) \ge \frac{\phi ^{\gamma }}{Z^{k-1}}$, where $\gamma = \sum _{j=1}^{k-i}j$ and *Z* is the normalizing factor for a Mallows model.

#### Proof

In order for $h_{i}$ to be available, there need to be $r'_{i+1},\ldots ,r'_{k}$ with preference orders $\eta _{i+1},\ldots ,\eta _{k}\in D ^{\phi ,\sigma }$ such that they were assigned hospitals $h_{i+1},\ldots ,h_{k}$. Hence, at the very least, $h_{i+1}\succ _{\eta _{i+1}}h_{i},\ldots ,h_{k}\succ _{\eta _{k}}h_{i}$. According to Lemma 3, the probability for each of these events is at most $\frac{\phi }{Z},\ldots ,\frac{\phi ^{k-i}}{Z}$ (respectively). Since they are independent of each other, and since the maximum probability for any particular $\eta \in D ^{\phi ,\sigma }$ is $\frac{1}{Z}$, the probability of a particular preference set occurring in which $h_{i}$ is available is at least $\frac{\phi ^{\gamma }}{Z^{k-1}}$. $\square$

#### Proof

(Theorem 8) Looking at the condition of Lemma 5 (recall $S^\prime = \{h_{1},\ldots ,h_{k}\} {\setminus } \{h_j\} \cup \{h_{k+1}\}$ for any $h_{j}\in \{h_{1},\ldots ,h_{k}\}$)

$$\begin{aligned} P(h_j )\mathbb {E}(v(h_j) | D ^{\phi ,\sigma })&\ge P(h_j ) \mathbb {E}(v(h_{k+1}) | D ^{\phi ,\sigma }) \\&+ \sum _{\eta \in H_\succ } P(\eta | D ^{\phi ,\sigma }) \Bigg [\sum _{h_i \in S^\prime } P(h_i )\mathbb {1}(h_{k+1} \succ _\eta h_i)v(h_{k+1},\eta ) \Bigg ] \end{aligned}$$

We again begin by expanding the value expectation ($\mathbb {E}$). This can be divided into *n* different inequalities:

$$\begin{aligned} P(h_j )P(h_{j}{\text { in }}\,s_{1})v(s_{1})\ge&v(s_{1})\Bigg [P(h_j )P(h_{k+1}{\text { in }}\,s_{1})\\ &+ \sum _{\begin{array}{c} \eta \in H_\succ |\\ h_{k+1}{\text { in }}\, s_{1} \end{array}} P(\eta | D ^{\phi ,\sigma })\sum _{h_i \in S^\prime } P(h_i )\mathbb {1}(h_{k+1} \succ _\eta h_i)\Bigg ]\\ \vdots \\ P(h_j )P(h_{j}{\text { in }}\,s_{n-1})v(s_{n-1})\ge&v(s_{n-1})\Bigg [P(h_j )P(h_{k+1}{\text { in }}\,s_{n-1})\\&+\sum _{\begin{array}{c} \eta \in H_\succ |\\ h_{k+1}{\text { in }}\, s_{n-1} \end{array}} P(\eta | D ^{\phi ,\sigma })\sum _{h_i \in S^\prime } P(h_i )\mathbb {1}(h_{k+1} \succ _\eta h_i)\Bigg ]\\ P(h_j )P(h_{j}{\text { in }}\,s_{n})v(s_{n})\ge&v(s_{n})P(h_j )P(h_{k+1}{\text { in }}\,s_{n}) \end{aligned}$$

We shall show that under the theorem’s assumptions, none of these inequalities hold for $h_{1}$, and therefore the general inequality (Lemma 5) does not hold.

Note that for each inequality we can simply ignore $v(s_{\ell })$ ($1\le \ell \le n$), since they appear on both sides of the inequality. The assumption of the theorem, since we are using plurality, is that the first inequality does not hold, i.e.,

$$\begin{aligned} P(h_1 )P(h_{1}{\text { in }}\,s_{1})&< P(h_1 )P(h_{k+1}{\text { in }}\,s_{1}) \\&\quad + \sum _{\begin{array}{c} \eta \in H_\succ |\\ h_{k+1}{\text { in }}\, s_{1} \end{array}} P(\eta | D ^{\phi ,\sigma })\sum _{h_i \in S^\prime } P(h_i )\mathbb {1}(h_{k+1} \succ _\eta h_i) \end{aligned}$$

As noted in Observation 1 (end of Sect. 3), for any $1<\ell \le k$ the probability of $h_{1}$ being in any spot $s_{\ell }$ is monotonically decreasing with $\ell$, while the probability of $h_{k+1}$ being in spot $s_{\ell }$ is monotonically increasing with $\ell$. Hence, $P(h_1 )P(h_{1}{\text { in }}\,s_{1})>P(h_1 )P(h_{1}{\text { in }}\,s_{\ell })$.

Similarly, $P(h_1 )P(h_{k+1}{\text { in }}\,s_{1})<P(h_1 )P(h_{k+1}{\text { in }}\,s_{\ell })$. We analogously see that:

$$\begin{aligned} \sum \limits _{\begin{array}{c} \eta \in H_\succ |\\ h_{k+1}{\text { in }}\, s_{1} \end{array}} P(\eta | D ^{\phi ,\sigma })&\sum \limits _{h_i \in S^\prime } P(h_i )\mathbb {1}(h_{k+1} \succ _\eta h_i)<\\&\sum \limits _{\begin{array}{c} \eta \in H_\succ |\\ h_{k+1}{\text { in }}\, s_{\ell } \end{array}} P(\eta | D ^{\phi ,\sigma })\sum \limits _{h_i \in S^\prime } P(h_i )\mathbb {1}(h_{k+1} \succ _\eta h_i) \end{aligned}$$

Simply put, the LHS gets smaller, while the RHS increases. Hence, for $1\le \ell \le k$:

$$\begin{aligned} P(h_1 )P(h_{1}{\text { in }}\,s_{\ell })&< P(h_1 )P(h_{k+1}{\text { in }}\,s_{\ell })\\&\quad + \sum _{\begin{array}{c} \eta \in H_\succ |\\ h_{k+1}{\text { in }}\, s_{\ell } \end{array}} P(\eta | D ^{\phi ,\sigma })\sum _{h_i \in S^\prime } P(h_i )\mathbb {1}(h_{k+1} \succ _\eta h_i) \end{aligned}$$

By Observation 1, for any $\ell >k$, $P(h_{1}{\text { in }}\,s_{\ell })<P(h_{k+1}{\text { in }}\,s_{\ell })$ which gives us:

$$\begin{aligned} P(h_1 )P(h_{1}{\text { in }}\,s_{\ell })&< P(h_1 )P(h_{k+1}{\text { in }}\,s_{\ell }) \implies \\ P(h_1 )P(h_{1}{\text { in }}\,s_{\ell })&< P(h_1 )P(h_{k+1}{\text { in }}\,s_{\ell })+\\ &+ \sum _{\begin{array}{c} \eta \in H_\succ |\\ h_{k+1}{\text { in }}\, s_{\ell } \end{array}} P(\eta | D ^{\phi ,\sigma })\sum _{h_i \in S^\prime } P(h_i )\mathbb {1}(h_{k+1} \succ _\eta h_i) \end{aligned}$$

Starting with the assumption that assortative interviewing does not hold for plurality, we show that none of the inequalities above hold for any slot $s_\ell$, and therefore that the condition in Lemma 5 does not hold for $j=h_{1}$ for any valuation function. $\square$

#### Proof

(Theorem 10) By Theorem 8, if assortative interviewing is not an equilibrium for plurality due to $h_{1}$, it is never an equilibrium for any scoring rule. If we compute the marginal contribution from some $h^* \in \{h_1,h_2,h_3,h_4\}$, and the contribution from $h^*$ is strictly less than the contribution from $h_5$ for any $\phi$, assortative interviewing is not an equilibrium for $k = 4$ and plurality. We find that the contribution from $h_1$ is less than the marginal contribution from $h_5$.

To calculate $P(h_1 )$, we simply iterate over all six possible allocations for $r_1, r_2, r_3$ such that $h_1$ is not taken, and directly calculate the probabilities of each ranking profile for $r_1, r_2, r_3$ that allows that to happen. In the interest of clarity, we only provide a symbolic representation. Let a permutation of $h_{2},h_{3},h_{4}$ be denoted as $(a_{1},a_{2},a_{3})$, and let *A* be the set of all such permutations (i.e., $(a_{1},a_{2},a_{3})\in A$ is a particular permutation of $h_{2},h_{3},h_{4}$).

$$\begin{aligned}&P(h_1 ) = \\&\sum _{(a_1,a_2,a_3) \in A} P(\mu (r_1) = a_1)P(\mu (r_2) = a_2 | \mu (r_1) = a_1)P(\mu (r_3) = a_3 | \mu (r_1) = a_1, \mu (r_2) = a_2) \end{aligned}$$

We instantiate the above equation using the probabilities of each potential match, and use numerical methods to show the function $P(h_1 ) - \phi ^4$ is negative for any $\phi$ in $0< \phi < 1$. $\square$

#### Proof

(Theorem 9)

Due to Theorem 8, it is enough to show there is no assortative equilibrium under plurality (and that $h_{1}$ violates Lemma 5’s condition). We use the simplification from Lemma 6: $P(h_j ) \ge \phi ^{k-j+1}$, and we will show it does not hold. Appealing to Lemma 9, we know $P(h_{j})$ is of the form:

18 $$\begin{aligned} P(h_{j})=\frac{X(k)}{Z^{k-1}}\phi ^{\sum \limits _{i=1}^{k-j}i}+\frac{X^{1}(k)}{Z^{k-1}}\phi ^{1+\sum \limits _{i=1}^{k-j}i}+\cdots + \frac{X^{\ell }(k)}{Z^{k-1}}\phi ^{(k\sum \limits _{i=1}^{k-j}i)-1}+ \frac{1}{Z^{k-1}}\phi ^{k\sum \limits _{i=1}^{k-j}i} \end{aligned}$$

($X(k), X^{1}(k),\ldots ,X^{\ell }(k)$ are functions that calculate the number of different sets of possible preference orders for $r_{1},\ldots , r_{k}$, with each set being a particular distance from the ground truth $\sigma$, thus having the probability $\phi ^{\sum _{i=1}^{k-j}i}$ for $X(k),\phi ^{1+\sum _{i=1}^{k-j}i} {\text { for }}\,X^{1}(k),$ etc.)

When $\phi \rightarrow 0$, $Z^{k-1}\rightarrow 1$, Eq. 18 becomes $P(h_{j})\rightarrow X(k)\phi ^{\sum _{i=1}^{k-j}i}$. In particular, there is $\varepsilon '$, such that $P(h_{1})<X(k)\phi ^{(\sum _{i=1}^{k-j}i)-1}$, and there is $\varepsilon =\min (\varepsilon ',\frac{1}{X(k)})$ such that for $\phi <\varepsilon$, for $k>3$:

$$ \phi ^{k-j+1}\ge \phi ^{k}\ge \phi ^{\left( \displaystyle \sum \limits _{i=1}^{k-j}i\right) -2}> X(k)\phi ^{\left( \displaystyle \sum \limits _{i=1}^{k-j}i\right) -1}>P(h_{1}) $$

This contradicts the condition stated in Lemma 6. $\square$

## Proofs from Sect. 6

### Proof

(**Theorem** 11) We look at the top *k* hospitals—$h_{1}, \ldots ,h_{k}$. If the same *k* residents interview in all of them (and none others do), these are strongly/pseudo-strongly assortative, and all the top *k* hospitals are taken, and we can move on and ignore these *k* hospitals (and the *k* residents interviewing there) until we find the first set of *k* hospitals that do not all interview strongly/pseudo-strongly assortative. For notational simplicity, let us assume that $r_{1}, \ldots ,r_{k}$ are not strongly assortative with hospitals $h_{1},\ldots ,h_{k}$. Hence, at least one of $r_{1},\ldots ,r_{k}$ interviews at $\{h_j, h_{j+1}, \ldots , h_{j+k-1}\}$ for some $2\le j\le n-k+1$. Thus, the number of residents from $r_{1},\ldots ,r_{k}$ interviewing at $h_{1}$ is smaller than *k*.

- If the number of interviewees at $h_{1}$ is $>k$, this means more than *k* residents are interviewing at the top *k* hospitals—$h_{1},\ldots ,h_{k}$—so at least one of these residents will not get a hospital.
- If the number of interviewees at $h_{1}$ is exactly *k*, recall that for this to be a weak assortative equilibrium there is a resident in $r_{1},\ldots ,r_{k}$ that is not interviewing at $h_{1}$ but rather interviews at $h_{j},\ldots ,h_{j+k-1}$ for $2\le j\le k$. Thus, there is a positive probability that this resident will prefer hospitals from the top *k* hospitals, and will get one of them. However, since *k* residents are interviewing at $h_{1}$, we know that in addition to the resident that does not interview there, there are *k* residents interviewing at $h_{1}$, i.e,. at the top *k* hospitals (since this an assortative strategy, anyone interviewing at $h_{1}$ interviews at $h_{1},\ldots ,h_{k}$). Since one of the top *k* hospitals is taken by the resident not interviewing at $h_{1}$, there are not enough hospitals for the *k* residents interviewing at $h_{1},\ldots ,h_{k}$, leaving at least one without a hospital.
- If the number of interviewees at $h_{1}$ is $<k$ but larger than 1, let us examine the resident with the highest index that interviews at $h_{1}$, which we shall denote $\bar{r}$. We examine this resident’s choices:
  - If there is no positive probability that any hospital $h_{1},h_{2},\ldots ,h_{k}$ is available, $\bar{r}$ will be left without any hospital.
  - If there is a positive probability that $h_{1}$ and some other hospital $h_{i}$ ($2\le i\le k$) are both available to $\bar{r}$, there is a possibility $\bar{r}$ prefers $h_{i}$, and thus there is positive probability $h_{1}$ will have no resident that choses to go there. Since we have *n* residents and *n* hospitals, if $h_{1}$ has no resident, there is a resident without a hospital.
  - If there is only a positive probability that $h_{1}$ is available and no $h_{i}$ ($2\le i\le k$) has any positive probability of being available, it means that even if the weakly-assortative resident in $r_{1},\ldots ,r_{k}$ that does not interview at $h_{1}$ chooses to go to some $h_{j}$, $j>k$, there is still no alternative hospital that will be available for $\bar{r}$ to choose from if $h_{1}$ is available. But this means that there is a choice for each resident before the one we analyze that gets a hospital, leaving only $h_{1}$ available, i.e., a previous resident interviewing at $h_{1}$ chose some hospital $h_{j'}\in \{h_{2},\ldots ,h_{k}\}$. Therefore, there is a probability that the residents’ preferences are such that the previous agent interviewing at $h_{1}$ chose $h_{1}$, and the weakly-assortative resident that chose $h_{j}$ can chose $h_{j'}$, and no other residents’ choices change, leaving our resident without any available hospital.
- If the number of interviewees at $h_{1}$ is exactly 1, if this resident does not choose $h_{1}$ but some other hospital, we have $n-1$ residents (everyone except the one interviewing at $h_{1}$) interviewing at $n-2$ hospitals (all hospitals except $h_{1}$, which they didn’t interview at, and the one our resident chose), ensuring some will not find a hospital. If we assume the only resident interviewing at $h_{1}$ chose $h_{1}$, we can simply ignore this resident and the hospital $h_{1}$ and repeat this proof with $n-1$ agents and hospitals.

$$\square $$

### Proof

(Theorem 12) Recall that agents wish to maximize their expected utility. Beyond the probability of ranking each hospital over another (which depends on the ground truth $\sigma$ and $\phi$), this utility also depends on each hospital’s availability based on the previous residents’ choices. For example, when interviewing in a set of hospitals only one of which is free (e.g., when $r_{1}$ interviews at $h_{1}$ and $h_{2}$, if $r_{2}$ interviews at $h_{1}$ and $h_{2}$, $r_{2}$ will get the hospital $r_{1}$ did not choose), the resident’s utility when interviewing at set *S* of hospitals is $\sum _{h\in S}P(h )E(v(h))$. Therefore, we need to keep in mind the probability of hospitals being available, which changes following each resident’s interviews, while the expected value from each hospital is the same for every resident (as they are sampled from the same distribution).

Resident $r_{1}$ will always bid on $h_{1}$ and $h_{2}$. If $r_{2}$ does as well, then we have a strong assortative equilibrium. Similarly, if $r_{2}$ interviews $h_{3}$ and $h_{4}$, $r_{3}$ would interview at $h_{1}$ and $h_{2}$ (since $\phi <1$, $h_{1}$ and $h_{2}$ are preferable to $h_{3}$ and $h_{4}$ as well as to $h_{2}$ and $h_{3}$), hence $r_{4}$ would interview at $h_{3}$ and $h_{4}$ resulting in a pseudo-strong equilibrium. Hence, in order to have at least one resident with a weak assortative strategy, $r_{2}$ interviews at $h_{2}$ and $h_{3}$. Before starting the analysis of $r_{3}$ let us note the probability of each hospital being available after $r_1$ and $r_2$ have chosen their interviews.

$$h_{1}$$: $$\frac{\phi }{1+\phi }$$ (i.e., $$r_{1}$$ choosing $$h_{2}$$).
$h_{2}$: $\frac{1}{1+\phi }\frac{\phi }{1+\phi }=\frac{\phi }{(1+\phi )^{2}}$ (i.e., $r_{1}$ choosing $h_{1}$ and $r_{2}$ choosing $h_{3}$).
$h_{3}$: $\frac{1}{1+\phi }\frac{1}{1+\phi }=\frac{1}{(1+\phi )^{2}}$ (i.e., $r_{1}$ choosing $h_{1}$ and $r_{2}$ choosing $h_{2}$).
$h_{4}$: 1 (since neither $r_{1}$ or $r_{2}$ interview there, $h_{4}$ will not be taken by them).

The third resident, $r_{3}$ may interview in $h_{1}$ and $h_{2}$, $h_{2}$ and $h_{3}$ or $h_{3}$ and $h_{4}$. We first show that $r_3$ will not interview in $h_{1}$ and $h_{2}$. We have:

$$\begin{aligned} u_{r_{3}}(h_{1},h_{2})&=\frac{1}{1+\phi }\frac{\phi }{1+\phi }E(v(h_{2}))+\frac{\phi }{1+\phi }E(v(h_{1})) \end{aligned}$$

This is because $r_{3}$ will get $h_{2}$ if $r_{1}$ chooses $h_{1}$ and $r_{2}$ chooses $h_{3}$, while $r_{3}$ will get $h_{1}$ if $r_{1}$ goes for $h_{2}$.

Interviewing in $h_{1}$ and $h_{3}$ (which is not weakly assortative) has the utility:

$$\begin{aligned} u_{r_{3}}(h_{1},h_{3})&=\frac{1}{1+\phi }\frac{1}{1+\phi }E(v(h_{3}))+\frac{\phi }{1+\phi }E(v(h_{1})) \end{aligned}$$

If interviewing in $h_{1}$ and $h_{2}$ is preferable, these equations imply $E(v(h_{3}))<\phi E(v(h_{2}))$. However, simple analysis shows that this is impossible: the ratio of expected values of consecutive hospitals cannot be less than $\phi$ (the ratio $\frac{E(v(h_{3}))}{E(v(h_{2}))}$ is exactly $\phi$ when the scoring function is plurality; anything that gives non-zero values to hospitals not in first place will make the ratio higher than $\phi$). So interviewing in $h_{1}$ and $h_{2}$ cannot be a strategy in an equilibrium. Furthermore, as is clear from the availability probabilities, since the probability of $h_{1}$ being available is higher than that of $h_{2}$, it makes no sense to interview in $h_{2}$ and $h_{3}$ and not in $h_{1}$ and $h_{3}$ (the resident increases their chance of getting a better hospital than $h_{3}$ by interviewing in $h_{1}$).

If all agents are weakly assortative, this leaves the case of $r_{3}$ interviewing in $h_{3}$ and $h_{4}$.^8^ Once again, let us look at the probability each hospital is available for $r_{4}$:

Before starting the analysis of $r_{3}$ let us note the probability of each hospital being available:

$$h_{1}$$: $$\frac{\phi }{1+\phi }$$ (i.e., $$r_{1}$$ choosing $$h_{2}$$).
$h_{2}$: $\frac{1}{1+\phi }\frac{\phi }{1+\phi }=\frac{\phi }{(1+\phi )^{2}}$ (i.e., $r_{1}$ choosing $h_{1}$ and $r_{2}$ choosing $h_{3}$).
$h_{3}$: $\frac{1}{1+\phi }\frac{1}{1+\phi }\frac{\phi }{1+\phi }=\frac{\phi }{(1+\phi )^{3}}$ (i.e., $r_{1}$ choosing $h_{1}$, $r_{2}$ choosing $h_{2}$, and $r_{3}$ choosing $h_{4}$).
$h_{4}$: $\frac{1}{1+\phi }\frac{1}{1+\phi }\frac{1}{1+\phi }=\frac{1}{(1+\phi )^{3}}$ (i.e., $r_{1}$ choosing $h_{1}$, $r_{2}$ choosing $h_{2}$, and $r_{3}$ choosing $h_{3}$).

This situation can be viewed graphically in Fig. 1. The availability of $h_{1}$ is better than that of $h_{2}$ and $h_{3}$ for any $\phi$, and therefore, it will always make sense interviewing at $h_{1}$ over $h_{2}$ and $h_{3}$. So if any interview set is chosen that does not include $h_{1}$, it is beneficial to swap one of those for $h_{1}$. We now need to show that $r_{4}$ interviewing with $h_{1}$ and $h_{2}$ (which is the only weakly assortative strategy that includes $h_{1}$) is not optimal.^9^

It is clear that if $E(v(h_{2}))$ is close in value to $E(v(h_{4}))$ then $h_{1}$ and $h_{4}$ have a higher expected utility than $h_{1}$ and $h_{2}$, thanks to $h_{4}$’s greater probability of being available. Notice that $r_{4}$’s utility from $h_{1}$ and $h_{2}$ is the same as that of $r_{3}$ with those same hospitals, and $r_{3}$’s strategy indicates that it is preferable to chose $h_{4}$ and not interview at $h_{1}$ or $h_{2}$ at all. If $E(v(h_{4}))$ was small compared to $E(v(h_{2}))$ (and thus compared to $E(v(h_{1}))$ as well), $r_{3}$’s choice of $h_{4}$ would not have happened. Analytical tools can be used to verify that $r_{4}$’s choice is either $h_{1}$ and $h_{4}$ (so not weakly assortiative), or $r_{3}$ would not have interviewed at $h_{3}$ and $h_{4}$ (leading to the problems detailed above for $r_{3}$, and hence not be weakly assortiative). $\square$

## Footnotes

1 Lee and Schwarz (2017) model includes interview costs and variable interviewing sizes, which our model avoids for simplicity. Adding them is discussed in the conclusion.

2 The assumption that there are an equal number of residents and hospitals is without loss of generality. If there are more residents than hospitals, then the lowest ranked residents will not obtain any interviews and can therefore be ignored. If there are more hospitals than residents, we can add “dummy” residents having the lowest ranks and the matching mechanism can ignore the match of any dummy resident.

3 Independently, Kadam (2015) provides a proof of a pure equilibrium similar to ours, despite the differing models, as both proofs rely on the serial structure of the deferred-acceptance algorithm.

4 We are assuming for convenience that *k* divides *n*. When *k* does not divide *n*, there will be some remaining $k' < k$ residents that will interview with the remaining $k'$ hospitals: $h_{\lfloor \frac{n}{k}\rfloor k+1},\ldots ,h_{n}$.

5 This is also known as Impartial Culture, a term introduced in Garman and Kamien (1968).

6 The ordering of the hospitals is the residents’ expected ordering. This would, for example, coincide with the reference ranking in the Mallows model.

7 Though note that in an equilibrium, the number of interviews in each hospital will still be *k*, for the reasons outlined following Theorem 12 when the cost for residents of being left without a hospital is high.
